# An Information Theory-Inspired Strategy for Design of Re-programmable Encrypted Graphene-based Coding Metasurfaces at Terahertz Frequencies

**DOI:** 10.1038/s41598-018-24553-2

**Published:** 2018-04-18

**Authors:** Ali Momeni, Kasra Rouhi, Hamid Rajabalipanah, Ali Abdolali

**Affiliations:** 1 Department of Electrical Engineering, Iran University of Science and Technology, Tehran, 1684613114 Iran; 2 Applied Electromagnetic Laboratory, School of Electrical Engineering, Iran University of Science and Technology, Tehran, 1684613114 Iran

## Abstract

Inspired by the information theory, a new concept of re-programmable encrypted graphene-based coding metasurfaces was investigated at terahertz frequencies. A channel-coding function was proposed to convolutionally record an arbitrary information message onto unrecognizable but recoverable parity beams generated by a phase-encrypted coding metasurface. A single graphene-based reflective cell with dual-mode biasing voltages was designed to act as “0” and “1” meta-atoms, providing broadband opposite reflection phases. By exploiting graphene tunability, the proposed scheme enabled an unprecedented degree of freedom in the real-time mapping of information messages onto multiple parity beams which could not be damaged, altered, and reverse-engineered. Various encryption types such as mirroring, anomalous reflection, multi-beam generation, and scattering diffusion can be dynamically attained via our multifunctional metasurface. Besides, contrary to conventional time-consuming and optimization-based methods, this paper convincingly offers a fast, straightforward, and efficient design of diffusion metasurfaces of arbitrarily large size. Rigorous full-wave simulations corroborated the results where the phase-encrypted metasurfaces exhibited a polarization-insensitive reflectivity less than −10 dB over a broadband frequency range from 1 THz to 1.7 THz. This work reveals new opportunities for the extension of re-programmable THz-coding metasurfaces and may be of interest for reflection-type security systems, computational imaging, and camouflage technology.

## Introduction

Metasurfaces, as two-dimensional (2D) artificial structures, overcome the limitations associated with their three-dimensional (3D) counterparts, while their interactions with electromagnetic (EM) waves are still strong enough to provide unusual abilities from perfect absorption to wavefront shaping^1^. Recently, the concept of coding metasurfaces has emerged as a powerful wave manipulation tool in which, by exclusively arranging two anti-phase meta-atoms (metasurface bits), various coding patterns (metasurface bytes) are created, achieving unprecedented functionalities from microwave to terahertz (THz) spectra^2^. These structures have shown rapid acceleration since their creation, resulting in different but fixed operational statuses such as bending, focusing, steering, and diffusion-like scattering^3–8^. Regarding the Fourier relation between the coding layout and its radiated beams, diverse theorems and analytical tools in digital communication and information theory can serve for the design of coding metasurfaces, leading to various interesting functionalities. Lately, the concept of information theory has been inextricably linked to EM metasurfaces where Shannon’s entropy has been established to estimate the information contained in a coding metasurface^9,10^. Inspired by the information science, coding metasurfaces aim at recording information on arbitrarily directed radiated beams to further modulate the channel and enhance the information carrier. Nevertheless, multipath effects, EM interferences, or locating other detectors in the path of intended receivers can compromise the security somehow that the radiated beams containing information are allowed to be covertly intercepted. In this paper, the capability of convolutional encoders was borrowed to generate multiple parity beams via introducing a special type of phase-encrypted metasurface whose information cannot be simply eavesdropped or distorted. Among all functionalities governed by the coding metasurfaces, the diffusion-like far-field pattern comprising numerous parity beams pointing at random directions is believed to possess great potential applications from both practical and security viewpoints as it makes information encryption more secure^11–13^. However, the conventional brute-force optimizations to achieve diffusion-like far-field patterns cannot efficiently function for arbitrarily large metasurfaces anymore^14,15^.

THz radiation has emerged as a suitable platform to perform medical diagnostics^16^, imaging for security and material analysis^17^, and ultra-high data rate short-path wireless communications^18^. To perform far-field pattern encryption in a real-time manner, it is of great importance to design a re-programmable THz-coding metasurface whose functionality can be dynamically tuned. Recently, several studies have reported electronically controllable coding metasurfaces in the microwave frequency region using a pin-diode in each coding particle^19–21^. Nevertheless, as the frequency increases toward the THz frequency region, pin-diodes are not commercially available^22^ and the other tuning capabilities strongly depend on the nonlinear characteristics of the constitutive materials. Because of its outstandingly strong, supernaturally light, and electrically tunable features^23^, graphene material has many applications such as nanostructured THz antennas^24^, tunable absorbers^25,26^, and analog optical computing^27^. The Fermi energy level and thus graphene surface conductivity can be locally controlled using chemical doping or applying an external electrostatic voltage bias on graphene-insulator-semiconductor structures^28^. Thus, one can envision new opportunities for wave-matter interactions with re-programmable graphene-based coding metasurfaces in the THz spectrum.

This paper firstly proposes a new concept in which the THz coding metasurface can contribute to data transmission/reception regardless of the conventional digital communication with time-modulated signals. Through the proposed re-programmable coding metasurfaces, we could control the information sent by the transmitter in a real-time manner. A convolutional coding function is established to record an arbitrary information message onto a phase-encrypted coding metasurface, generating undetectable but identifiable parity beams pointing at certain directions. This issue can significantly enhance the capability of communication through creating parallel channels in addition to the traditional ones (i.e. one channel occupied with time-modulated signals carrying the information of the encoded message sequences and at the same time, another channel occupied with multiple parity beams carrying the information of the encoded message sequences). To implement such a platform, a single graphene-based reflective cell fed by dual-mode biasing voltages is also designed whose operational status can be dynamically switched between the two states of “0” and “1”. Armed with such a re-programmable device, the real-time mapping of information messages onto various protected far-field patterns generated by convolutionally encrypted metasurfaces is simply available. By targeting the maximum Renyi entropy of the generated parity beams, the optimal diffusion layout is extracted using a Binary Bat optimization algorithm to prove that the proposed convolutional encryption scheme convincingly offers a straightforward, reliable, and fast alternative design manner of diffusion coding metasurfaces of arbitrarily large size.

## Results

### Statistical study

**Figure 1:**
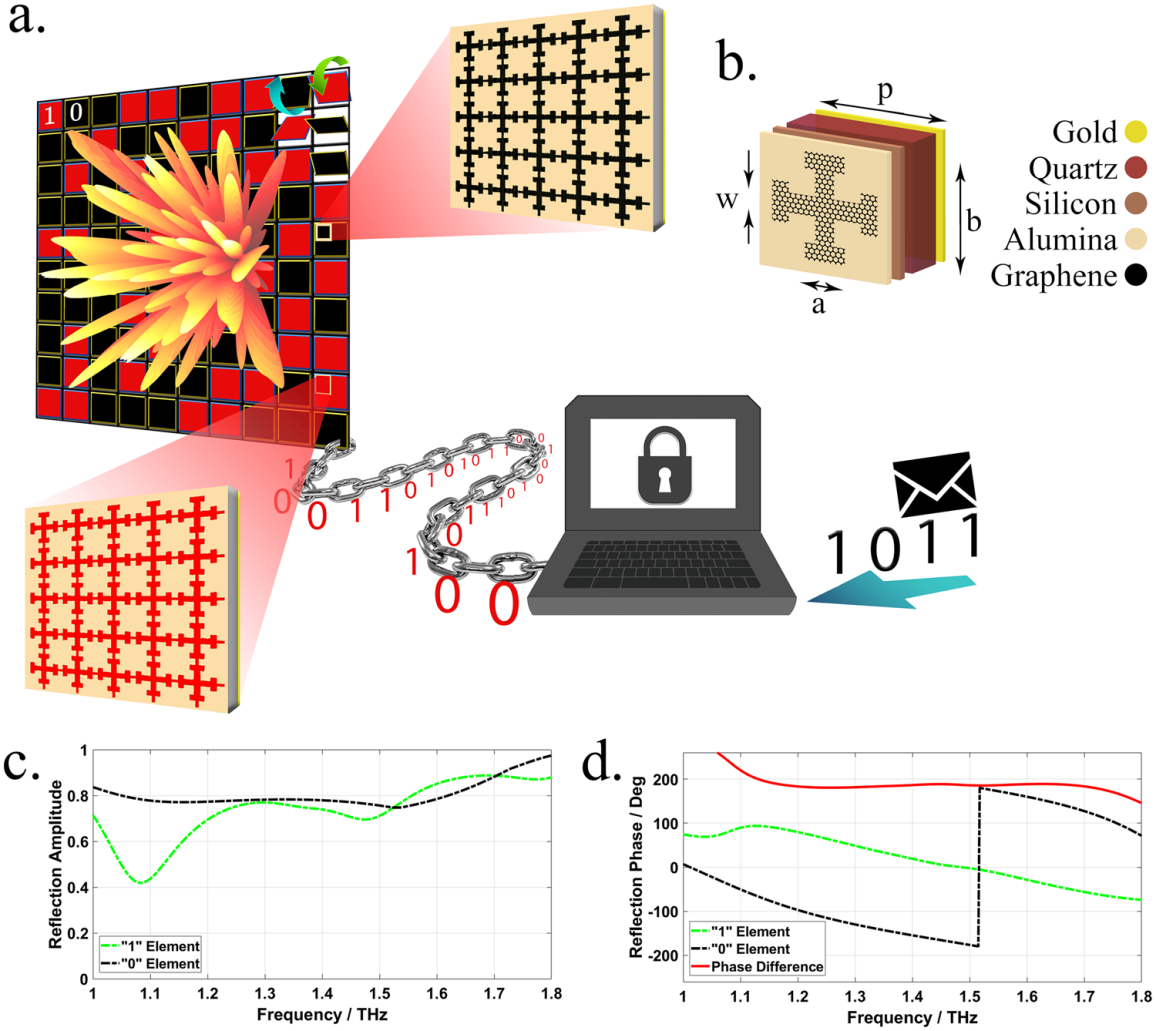
(**a**) The schematic diagram of the proposed reconfigurable THz coding metasurface including of N × N graphene-based digital supercells of the same size possessing different chemical potentials. The red and black colors refer to “0” and “1” basic elements, respectively. **(b)** The geometry of Jerusalem-shaped basic constitutive reflective cell. **(c)** The reflection amplitude spectra of the 0” and “1” digital meta-atoms. **(d)** The reflection phase spectra of the 0” and “1” digital meta-atoms.

Despite the interpretations governed by the generalized Snell’s law^29^, it is most suitable to discuss the far-field pattern from a statistical viewpoint to give a better understanding of the principal mechanism of diffusion metasurfaces. Herein, we set the framework for the statistical description of coding metasurfaces to provide the key concepts needed to obtain the optimal coding diffusion patterns. Consider a dynamically controllable coding metasurface schematically illustrated in Fig. 1a in which N × N equal-sized supercells (“0” and “1” digital metasurface bits) occupy the metasurface area. Without reducing the generality of concept, a 1-bit coding metasurface is considered here and the results can be simply extended to a multi-bit configuration. Hence, each supercell can instantaneously possess a reflection phase of either 0 or π when it is normally illuminated by a plane wave. In addition, each supercell is composed of M × M identical coding elements to minimize undesirable coupling effects between the neighboring supercells. To represent the binary coding patterns quantitatively, a 2D coding matrix can be utilized as follows:

1 $${\rm{M}}={(\begin{array}{ccc}1/0 & \cdots  & 1/0\\ \vdots  & \ddots  & \vdots \\ 1/0 & \cdots  & 1/0\end{array})}_{{\rm{N}}\times {\rm{N}}}$$

The spatial distribution of far-field patterns specifying the angular dependence of the scattered electric fields can be taken as a stochastic feature of a phase-encoded metasurface which can be described by evaluating their PDF_JP_ diagrams. Under a normal incidence, the far-field scattering pattern of a coding metasurface occupied with an arbitrary coding pattern can be characterized by a superposition from the contributions of all supercells^30^.

2-a $${E}_{scat}(\theta ,\phi )={E}_{elem}(\theta ,\phi )\times AF(\theta ,\phi )$$

2-b $$AF(\theta ,\phi )=\sum _{p=1}^{N}\sum _{q=1}^{N}{a}_{pq}\exp [j{k}_{0}D(p-1)sin\theta cos\phi ]\exp [j{k}_{0}D(q-1)sin\theta sin\phi ]\,\,$$

**Figure 2:**
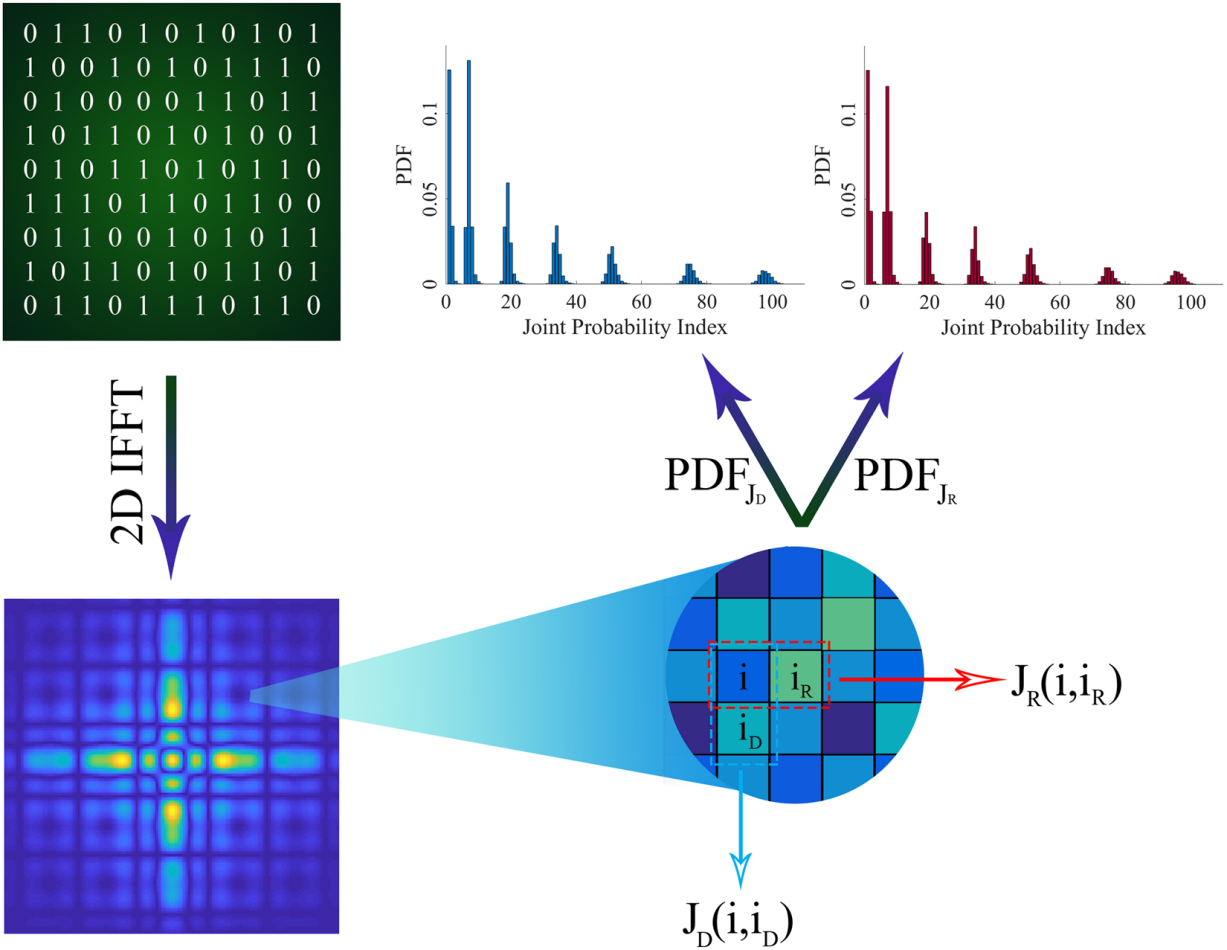
The employed procedure to compute the PDF_JP_ diagram from the IFFT-based far-field images for a specified coding matrix.

**Figure 3:**
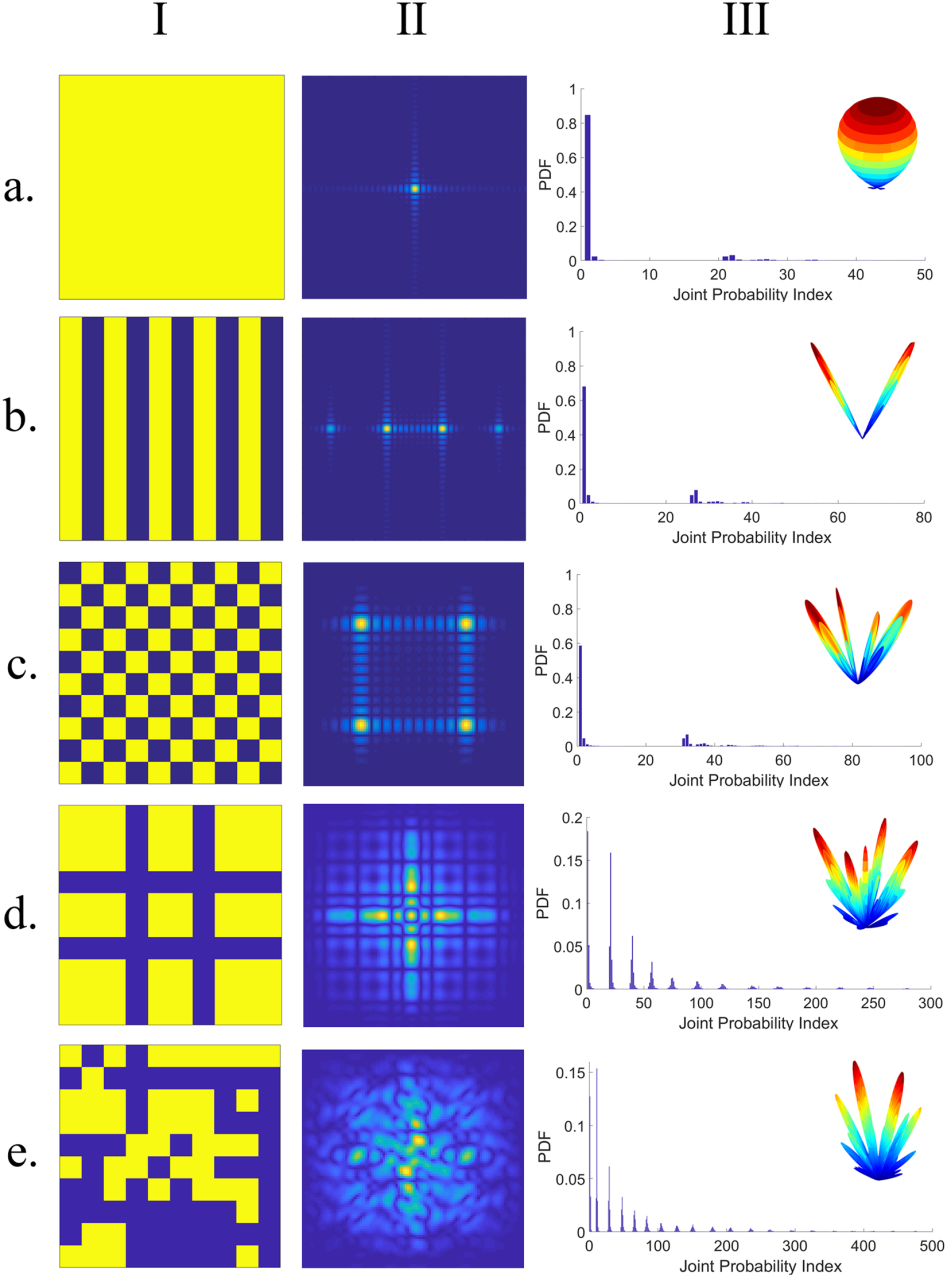
Differently encoded metasurfaces, their 2D far-field images and PDF_JP_ diagrams. (**a**) 11…1/11…1 (A-type or PEC reflector). **(b)** 1010…/1010… (B-type or stripped configuration). **(c)** 1010…/0101… (C-type or chessboard configuration). **(d)** Non-periodic configuration (D-type). **(e)** Arbitrary computer-generated pseudorandom configuration (E-type). (i) Coding patterns. (ii) 2D far-field images. (iii) PDF_JD_ diagrams of the far-field images.

Here, E_elem_(θ, φ) and a_pq_ = |a_pq_|exp(jφ_pq_) denote the far-field pattern function and the complex reflection coefficient of each supercell, respectively; D represents the period of elements along both x and y directions; k_0_ is the free-space wavenumber; and θ and φ denote polar and azimuthal angles, respectively. Due to the subwavelength property of meta-atoms, we can safely ignore E_elem_(θ, φ) during the far-field calculation. Moreover, the ground plane almost reflects the total energy to the upper half-space, thereby approaching a strong reflection amplitude for all supercells. By dramatically increasing the number of supercells in a large-scale coding metasurface, Equation 2 becomes computationally unaffordable. To circumvent this limitation, a 2D inverse fast Fourier transform (2D-IFFT) is adopted to significantly accelerate the far-field pattern prediction in the 2D polar coordinate system, which is very promising for our further investigations in this paper. The details about the manner of 2D-IFFT calculation can be found in Supplementary Information A. Owing to the existing connection between different coding patterns and corresponding radiated beams, our findings about the statistical features of far-field patterns would give insightful vision and contribute to a deeper understanding of the information carried by phase-encoded metasurfaces. Each phase-encoded metasurface is responsible for mapping the information contained in its coding layout onto a certain far-field pattern with the inverse Fourier transformation. The 2D far-field images resulting from the 2D-IFFT technique have a stochastic behavior and form a pseudorandom scattering matrix (S), comprising far-field pattern intensities. Based on different statuses of each neighboring pair of matrix elements in horizontal and vertical directions, one can define two PDF_JP_ diagrams of PDF_JR_ and PDF_JD_, respectively (see Fig. 2). Such stochastic parameters can be determined in a specific manner comprehensively discussed in Supplementary Information B. Without loss of generality, the coding patterns of 10 × 10 elements (N = 10) are only assumed here. Several phase-encoded metasurfaces are dedicated to accommodate different message sequences of 11…/11… (A-type coding metasurface as PEC mirror), 1010…/1010… (B-type coding metasurface with a periodic striped configuration), and 1010…/0101… (C-type coding metasurfaces with a periodic chessboard configuration) as the most popular representatives of the other coding layouts. Besides, two other D- and E-type metasurfaces are also constructed whose coding patterns contain a non-periodic and an arbitrary pseudorandom computer-generated information message, respectively. The resultant 2D IFFT-based far-field images, as well as 3D scattering patterns and the corresponding PDF_JP_ diagrams are depicted in Fig. 3. It should be noted that only the PDF_JD_ diagram is given here, and the horizontal one is shown in Supplementary Figure S1. As can be observed, each PDF_JP_ diagram generally consists of different numbers of sub-Gaussian components with soft peaks and short tails and super-Gaussian components with spiky peaks and long tails, having certain amplitudes. Evidently, the randomness of far-field patterns directly depends on the distribution of these components so that more uniform distribution would result in a more efficient diffusion-like far-field pattern, giving rise to numerous reflected beams. Indeed, as the randomness and complexity of the coding pattern determined by the information message increase, the number of sub-Gaussian and super-Gaussian components become greater. In this case, the far-field pattern information is difficult to be estimated since the amount of information recorded onto the far-field pattern is drastically increased on a pro rata basis. Additional details can be found in Supplementary Information B. In another sense, increasing the independency of adjacent supercells in such a randomized binary coding pattern results in a quasi-uniform distribution with a greater number of weak sub-Gaussian and super-Gaussian components (see Fig. 3e). Consequently, to describe the distribution of such statistical components in a quantitative manner, we should seek to find that of measure emphasizing on the Gaussianity of PDF_JP_ diagrams of the far-field patterns. Entropy is a key measure to interpret the expected value of the information contained in different shapes of far-field patterns. Obviously, the probability of eavesdropping or estimation of far-filed patterns is remarkably reduced when their average entropy measure as well as their diffusion level reaches maximum quantity. In particular, regarding the high ability to measure Gaussianity^31^, Renyi entropy can serve to intuitively describe different phase-encoded metasurfaces from an information viewpoint. By defining an entropy of order $\zeta \ne 0, > \,1$, the Renyi entropy (H_RE_) can be expressed as^32,33^

3 $${H}_{RE}=\frac{1}{1-\zeta }\,\mathrm{log}\,\sum _{i}{p}_{i}^{\zeta }$$

In the case of $\zeta =2$, the so-called quadratic Renyi entropy measure as ${H}_{RE}=-\,log{\sum }_{i}{p}_{i}^{2}$ equally emphasizes both sub-Gaussian and super-Gaussian components. Here, utilizing an average value of vertically and horizontally defined joint probability functions, the 2D quadratic Renyi entropy can estimate the information contained in 2D far-field images generated by differently arranged coding metasurfaces:

4 $${H}_{RE}=\frac{-1}{2}\,\mathrm{log}[\sum _{i=1}^{128}\sum _{{i}_{R}=1}^{128}{P}_{i{i}_{R}}^{2}+\sum _{i=1}^{128}\sum _{{i}_{D}=1}^{128}{P}_{i{i}_{D}}^{2}]$$

where, ${P}_{i{i}_{R}}$ and ${P}_{i{i}_{D}}\,\,$denote the elements of PDF_JR_ and PDF_JD_ diagrams which are indexed by ii_R_ and ii_D_, respectively. Here, relying on the ability of the Renyi entropy to detect sub- and super-Gaussian components, we investigate the far-field information contained in periodically, non-periodically, and randomly encoded metasurfaces, as shown in Fig. 3. According to the aforementioned procedure, the corresponding Renyi entropy values are computed and listed in Table 1. As expected, the far-field scattering patterns of regularly arranged metasurfaces of A-, B-, and C-types whose 0/1 coding patterns contain low uncertainty have small entropy values of 0.1430, 0.3178, and 0.4519, respectively. By increasing the complexity and the randomness of coding patterns from C- to D-type metasurfaces, the estimation probability of the far-field patterns apparently decreases, yielding a greater Renyi entropy value of 1.1342. A pseudorandom distribution of digital supercells deflects the incoming wave into many non-specular random directions, resulting in a diffuse scattering as one of the most prominent functionalities of THz coding metasurfaces. The far-field scattering pattern generated by such a random-coding metasurface (E-type) with an arbitrary pseudorandom computer-generated coding pattern has an entropy value larger than that of other configurations (1.3525). However, it still poorly acts as a diffusion metasurface. It seems that performing a proper information operation on the coding patterns would be greatly instrumental for further enhancing the far-field pattern information, the channel capacity, and the immunity of information against channel impairments.

**Table 1:** The computed Renyi entropy values for different configurations of a coding metasurface.

Type	Arrangement	Renyi entropy (H_RE_)
A	PEC reflector	0.1430
B	Stripped configuration	0.3178
C	Chessboard configuration	0.4519
D	Non-periodic configuration	1.1342
E	Arbitrary computer-generated pseudorandom configuration	1.3525

### Convolutionally encrypted metasurfaces

As coding metasurfaces are capable of producing various types of scattering patterns in the far-field domain, the information carried by generated beams can be tailored by designing diverse coding patterns with different information entropies. The carrier beams containing the information of messages are then transmitted into the free space, passing through the attenuated channel, facing the multipath effect and EM interference, and finally captured by several intended receivers (see Supplementary Figure S2). From the beginning, we should apply a simple and reversible encryption algorithm to the message sequences to create an encrypted coding pattern yielding a phase-encrypted metasurface and generate secure carrier beams providing a high degree of robustness against the channel impairments. To achieve this, inspired by digital communication, establishing the approach of error-correcting beams can be analogously issued to enhance the virtual channel capacity. In view of the existing duality between digital communication tools and coding metasurfaces, we propose a similar concept in which the message bits are convolutionally encoded and the parity bits are created as a function of all message bits. The parity bits at the output of this process form the encrypted coding pattern and so, the digital status of each supercell will be also encrypted, leading to a phase-encrypted metasurface. The coding metasurface driven by such an encrypted coding pattern generates an encrypted far-field pattern including a certain number of beams with different directions. Since the origin of the generation of these radiated beams is those parity bits forming the encrypted coding pattern, we name these beams, as *parity beams*. The parity beams of the coding metasurface have the same task that the parity bits do in the digital communication and similar features can be observed between them. Similar to wireless communication procedures, the parity beams generated by phase-encrypted metasurfaces are only sent over the channel instead of transmitting the far-field pattern belonging to the coding pattern fed by the original message.

**Figure 4:**
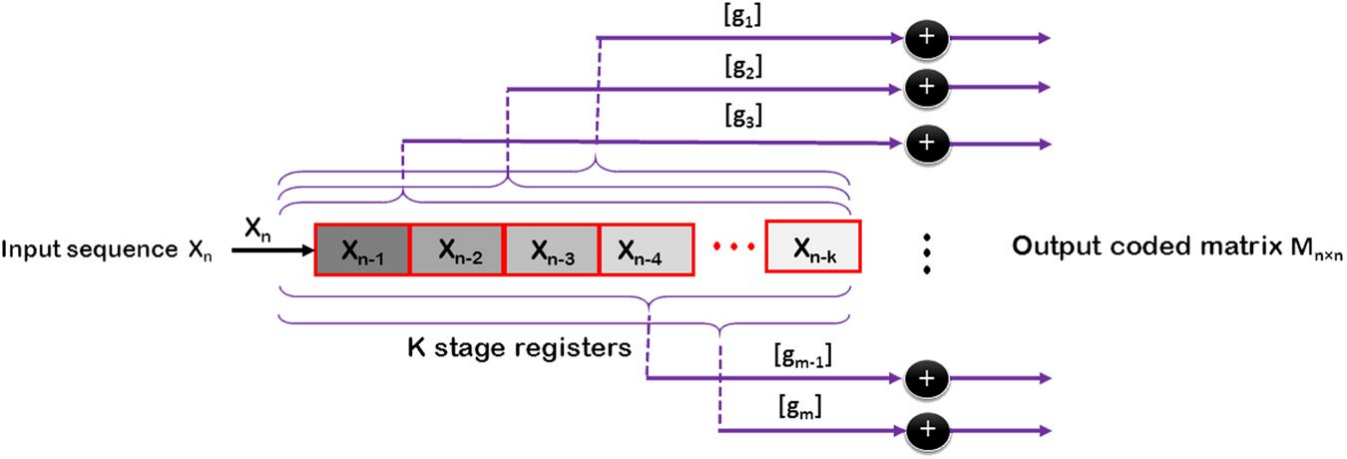
Block diagram view of the employed convolutional coding function to rapidly generate a semi-optimal diffusion coding pattern.

Let us consider an arbitrary *N*-bits binary message (*X*) whose original information is desired to be securely carried through multiple parity beams generated via a phase-encrypted metasurface of *N*^2^ supercells. The encrypted coding pattern can be extracted by convolving *X* with a specified encoder’s impulse response to illusory contain the information of the original digital message. The schematic diagram of the required convolutional phase-encoder fed by the *X* coding sequence is displayed in Fig. 4. The phase-encoder uses a sliding window with the size of *m* to calculate output bits by performing a module2addition or, equivalently, an exclusive-or operation on different subsets of input information bits in the window. Generally, each parity equation specifying the way that the encrypted bits are created from the information bits (*X*) is built by combining them and a generator polynomial, *g*. Each output bit *p*_*i*_ can be represented by a convolutional form of ^34,35^

5 $${p}_{i}[k]=(\sum _{j=0}^{N-1}{g}_{i}[j]x[k-j])\,mod2\,$$

With sliding a window on a certain input chain of *N* bits, a convolutional phase-encoder sequentially maps them to a *k*-bits randomized coding sequence (*k > N*), containing more information and thus a higher entropy level. The process is commonly specified with two essential parameters of code rate (*1/r*) and constraint length indicating the window size (*m*) as the encoder memory. First, *m* memory registers are initialized with zeros when a pre-defined information message of *N*-bits size (*X*) feeds them one after another. Obviously, different *N-*bits input-coding sequences would result in different *k-*bits encrypted codewords where an initial semi-irregular message becomes progressively a large and amorphous one. In our scheme, the re-programmable phase-encrypted metasurface has *N*^2^ digital supercells whose operational statuses can be dynamically switched between two states of ‘0’ or ‘1’. To obtain all required *N*^2^ elements of the 1/r = 1/N and the parameters of (*N*, *k*, *m*) = (*N*, *N*^2^, 5) should be adopted. The parity beams generated by such an encrypted coding pattern as the phase-encoder output bits, a convolutional coding function with the code rate of encrypted coding pattern carry the information contained in the original N-bits message in a secure manner. Each time, the coding metasurface is digitally programmed by an encrypted layout governed by the message sequences and the carrier beams continue to propagate until they are partially captured by several intended receivers. Each receiver antenna takes a sample from the far-field electric field intensities along a certain direction, thus creating S (sinθcosφ, sinθsinφ) as 2D polar scattering matrix. The quality and integrity of the reconstructed scattering matrix ($\tilde{{\rm{S}}}$) strongly depends on the number of receivers, their locations, the multipath effects and the EM interferences. If the intended receivers totally and correctly record the electric field intensities of the far-field region (i.e. $\tilde{{\rm{S}}}$(i, j) components are accurately and perfectly reconstructed), by applying the Fourier transform, one can safely disclose the coding pattern information (but in its encrypted form) from the captured $\tilde{{\rm{S}}}$(i, j) elements. However, in most real-life scenarios, the perfect and accurate restoration of $\tilde{{\rm{S}}}$(i, j) elements is not available. Afterwards, the original message sequence (in a decrypted form) can be thoroughly retrieved by applying a decoding function to the reconstructed coding pattern (see Supplementary Information C).

**Figure 5:**
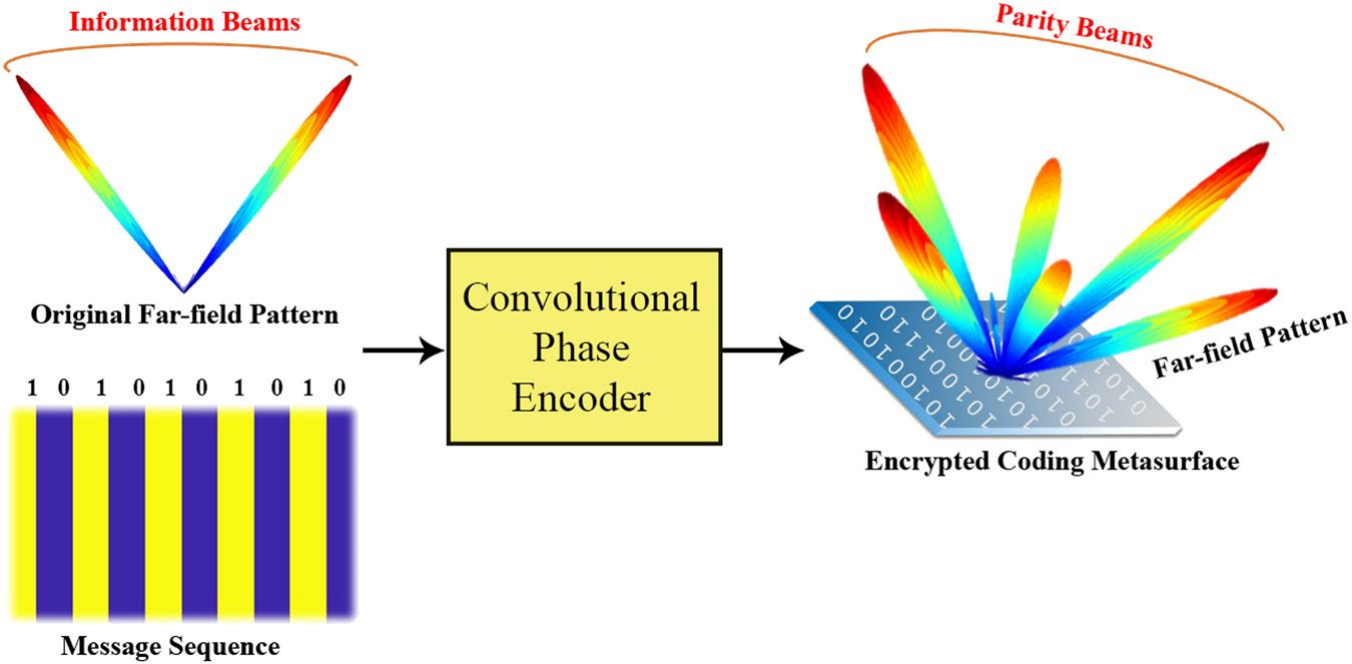
The convolutionally encrypted coding metasurfaces as secure information sources for generating multiple parity beams whose far-field pattern information cannot be easily distorted or estimated by the outsider receivers. Such phase-encrypted metasurfaces illusory record the information of the original message onto an undetectable but recoverable group of radiated parity beams.

To demonstrate the capability of the proposed encryption fashion, without loss of generality, a coding metasurface with 10 × 10 digital supercells (N = 10) and an arbitrary information message of *X* = {1010101010} is considered. In the first configuration, the information-coding sequence is directly written into the coding metasurface so that it is repeated in each row of the coding matrix^36^. Next, a phase-encoder with five memory registers is initialized with the aforementioned message. The windows overlap and slide by one to convolutionally create the encrypted coding pattern from the information message. Additional detail about the utilized generator polynomials can be found in Supplementary Table S1 of Supplementary Information D. According to the obtained coding pattern, the phase-encrypted metasurface is then constructed and the 3D far-field pattern is plotted in Fig. 5. The phase-encrypted coding metasurface (like an information source in digital communication) can generate multiple randomly directed parity beams (like parity bits in digital communication) containing the information of the desired message in a deceptive and secure manner. The corresponding Renyi entropy values are also given in Table 2. As can be seen, the striped coding metasurface filled by the original information message of 1010101010 provides two symmetrically oriented beams. The masked version of the coding metasurface as the phase-encrypted metasurface illusory radiates several randomly directed parity beams in appearance. Therefore, by comparing the Renyi entropy values, one can conclude that the information carried by the phase-encrypted metasurface is more than that of the stripped coding metasurface. Actually, the parity beams emitted by the encrypted metasurface contain the information of the original message, surreptitiously and with more uncertainty, such that they cannot be easily detected when they are captured by some outsider receivers.

**Table 2:** The computed Renyi entropy values for differently diffusion layouts.

Coding pattern	Renyi entropy (H_RE_)
Convolutionally encrypted-encoded with the input sequence of 1010101010	1.2174
Convolutionally diffusion-like encoded with the best random input sequence of 1010111101	1.6480
Optimal diffusion layout obtained by BBA algorithm	1.7946

### Encrypted diffusion metasurfaces

**Figure 6:**
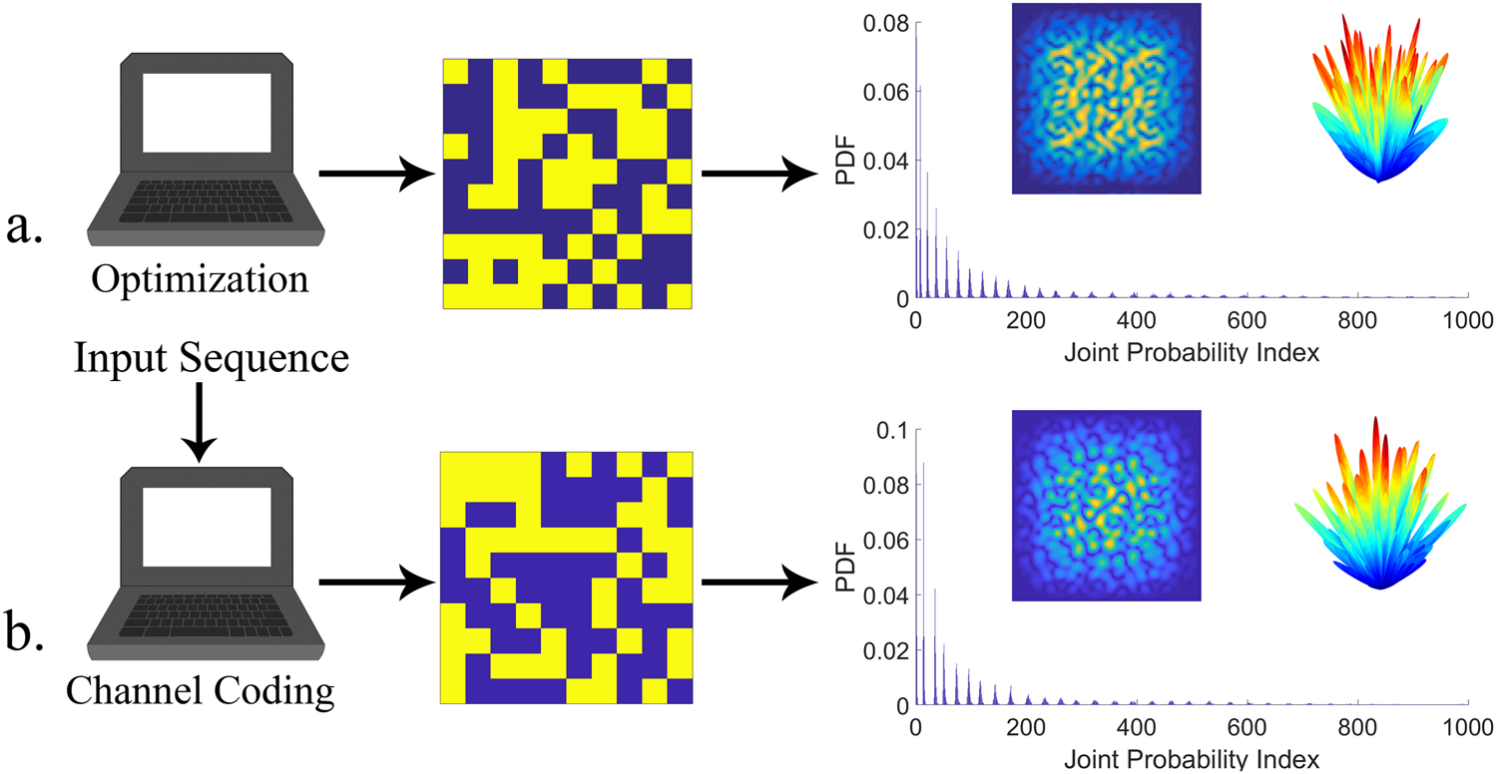
The PDF_JP_ diagrams and 2D far-field images for different diffusion coding patterns achieved by **(a)** Channel coding function **(b)** Binary Bat optimization algorithm.

In addition to the channel coding function, the security of the far-field pattern can be further guaranteed by the coding metasurface. Encrypting the information of message sequences onto a diffusion-like far-field pattern enables a more secure channel as it includes many parity beams along different random directions and thoroughly recording them by the outsider receivers is not a practical assumption. The trade-off, though, is that it needs a large-scale encrypted metasurface to perfectly map the information onto a diffusion-like far-field pattern having numerous parity beams. At the beginning, we aim to obtain the optimal diffusion layout among all ${2}^{{N}^{2}}$ possible solutions, which contains the maximum far-field pattern information. To achieve this, a Binary Bat optimization algorithm is adopted to ensure that the maximum Renyi entropy of Equation (4) becomes sufficiently large. As one of the novel heuristic optimization algorithms, the superior performance of this algorithm has been proven among other more well-known algorithms such as genetic algorithm (GA) and particle swarm optimization (PSO)^37,38^. More details can be found in Supplementary Information E. The far-field responses corresponding to the obtained optimum diffusion layout are illustrated in Fig. 6a. As can be observed, the Renyi entropy reaches its maximum value of as high as 1.7946. Consequently, the introduced optimization-based design algorithm achieves the desired diffusion-like scattering pattern comprising a large number of reflected beams with dramatic backscattering suppression. Although the optimized coding pattern is strongly random to deflect the incoming wave into numerous directions within the upper space, several pivotal open issues of sophistication, time-consuming procedures, and non-guaranteed global optimum solutions pertaining to the optimization algorithms pose a great difficulties for real-time designing of large-scale diffusion metasurfaces. Increasing the uncertainty in the number and direction of parity beams, ensures the far-field pattern information is not easily eavesdropped or distorted, as capturing them thoroughly by the outsider receivers is not a practical assumption. To meet this demand, security has to rely on an inherent entropy enhancement governed by the phase encryption of the metasurface via the convolutional phase encoder introduced in the previous section. Moreover, the convolutional encryption can greatly accelerate the process of randomization of the initial metasurface. In this case, the longer the constraint length, the larger the number of parity beams that are affected by an arbitrary information message. Since the parity beams are the only beams sent over the channel, a larger number of them implies a greater resilience to the channel impairments. In a sense, the re-writable diffusion metasurfaces of this paper can be a suitable platform to play the role of a secure information source for generating encrypted but identifiable groups of parity beams. In addition to reflection-type security systems, the achievement of arbitrarily large low-scattering surfaces can find important applications in other areas of imaging, camouflage technology, reflective dark-field microscopy measurements, and so on. Here, we seek to find a simple approach for gaining a convincing diffusion performance comparable with that expected from numerical optimizations such that it could be applied to any arbitrarily size metasurface with a low computational cost. A semi-optimal diffusion coding pattern storing maximum information entropy can be obtained via searching the best initial sequence of the convolutional encoder among all 2^N^ possible choices through a simple iteration cycle with a negligible computational burden (only a few minutes). The resultant far-field images are then informationally evaluated. It should be noted that a proper connection between the outputs and the memory registers (i.e. generator polynomials) should be adopted to convolutionally map the information of the input sequence to a crowded group of randomly directed parity beams. More details about the utilized generator polynomials can be found in Supplementary Table S2 of Supplementary Information D. For the sake of a better comparison, the same size of N = 10 is assumed and the corresponding far-field results are plotted in Fig. 6b. As can be seen, the diffusion performance of the best convolutionally encrypted metasurface is fairly comparable with that of the optimization-based coding pattern, creating numerous parity beams in random directions. Armed with such a randomized coding pattern, the phase-encrypted metasurface is capable of convincingly producing a diffusion-like scattering pattern having an entropy value as high as that provided by the numerically optimized diffusion metasurface. It should be noted that by exploiting the proposed fashion, the design problem is reduced to solely search the best N-bits input sequence of the encoder instead of finding the optimal diffuse coding pattern between all ${2}^{{N}^{2}}\,$possible situations. Despite conventional design approaches such as iterative optimization processes in CST and MATLAB software programs which take a long time, the proposed design strategy dramatically reduces time consumption and the required memory storage while convincingly preserving the diffusion performance. To demonstrate the high capability of the proposed encryption scheme, it is similarly applied to the coding metasurfaces of different sizes ($4\le N\le 100$). The corresponding results are given in Supplementary Information I. The satisfactory results elucidate a new insight into the application of information encryption for future designs of arbitrarily large diffusion metasurfaces, offering great potential applications in imaging, security systems, spectroscopy, etc. Besides, the rapid property of the proposed algorithm resolves to a great extent the major challenges reported in the design of large-scale diffusion metasurfaces.

### Graphene’s surface conductivity and digital meta-atoms

A reconfigurable coding metasurface with interchangeable functionalities at THz frequencies enables an unprecedented degree of freedom in real-time emission of the parity beams. Hereupon, relying on the prominent feature of graphene, as tunability, the beam manipulation capability of THz coding metasurfaces could be significantly enhanced. As assumed throughout this paper, the graphene-based coding metasurface is composed of 10 × 10 digital supercells arranged in a specified coding pattern, providing a certain but dynamically switchable operational status. Figure 1a depicts the details of the designed coding metasurface. To suppress the undesired effects caused by the partial interaction of adjacent supercells, they were occupied with 15 × 15 number of elaborately designed patterned graphene cells whereby two interchangeable opposite reflection phases of 0 and π are simply achieved through a dual-mode external electrostatic bias. Multi particles with different geometries or orientations are already involved in the realization of such coding metasurfaces, making the design procedure difficult and non-adaptable^39,40^. Conversely, our subwavelength graphene-based particles have the same size but different chemical potentials and can be arbitrarily adjusted to enable further facilitation of the design. The variation of the chemical potential of each cell while keeping its dimension unchanged, gives us plenty of degrees of freedom in reaching different functionalities at the same time, just by rearranging the coding patterns. The proposed unit cell contains a graphene-based Jerusalem cross metasurface at the top layer, ultrathin substrates of alumina and silicon providing biasing considerations at the second layer, a Quartz dielectric (ε_r_ = 3.75, tanδ = 0.0184) with the thickness of h = 25 μm at the third layer, and finally a gold $(\sigma =4.42\times {10}^{7}S/m)$ ground plane avoiding THz transmission at the bottom layer (see Fig. 1b). The periodicity of unit cells along the x and y directions are 16 μm and the other geometrical parameters are a = 5 μm, b = 13 μm, and w = 3 μm. All the reflective cells of a supercell are linked together through deep-subwavelength graphene ribbons whereby we can simply tune their chemical potentials with one electrostatic voltage instead of adjusting all the sub-units independently. Different chemical potentials of μ_C_ = 0.1 eV and μ_C_ = 1.16 eV are assigned to the meta-atoms to mimic the “0” and “1” digital particles of the coding metasurface, respectively. The previously reported results have experimentally proved the feasibility of such a structure within the realm of current fabrication technology^41,42^. More details about the external bias circuit of the graphene-based coding metasurface and the full-wave simulations are presented in Supplementary Information F. A field-programmable gate array (FPGA) hardware can be employed to generate the required bias voltages at its output terminals and change the operational status of each digital supercell, independently (see Supplementary Figure S4). A commercial software, CST Microwave Studio, was established to simulate the reflection amplitude and phase of the infinite array of meta-atoms, as depicted in Fig. 1c,d. From this figure one can conclude that the “0” element exhibits a zero reflection phase at 1 THz and its reflection phase inverses at 1.5 THz, while the near-zero reflection phase of “1” element is positioned in close proximity to same inverse frequency. Besides, the graphene-based metasurface provides high reflectivity across the frequency band of interest. Thus, a phase difference around 180° can be granted in a broad frequency range from 1.1 THz to 1.7 THz, leading to scattered energy cancellation in the normal direction.

**Figure 7:**
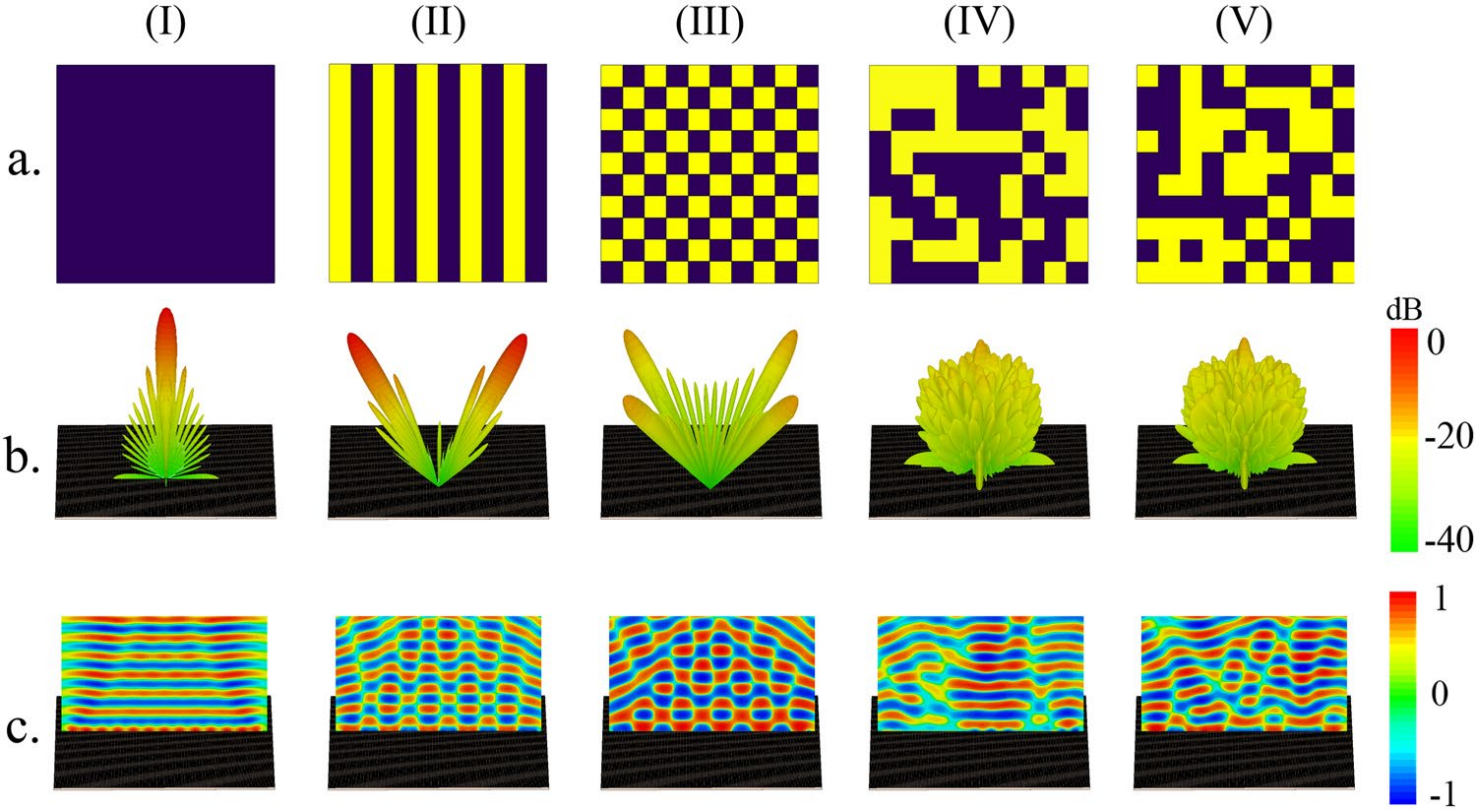
The 3D far-field patterns and in-plane patterns of the proposed reconfigurable coding metasurfaces. (**a**) Coding patterns. **(b)** 3D scattering patterns. **(c)** in-plane patterns. (i) 11…1/11…1 (A-type or PEC reflector). (ii) 1010…/1010… (B-type or stripped metasurface). (iii) 1010…/0101… (C-type or chessboard metasurface). (iv) Convolutionally encoded metasurface with the random input sequence of 1111010101. (v) The optimal diffusion metasurface.

**Figure 8:**
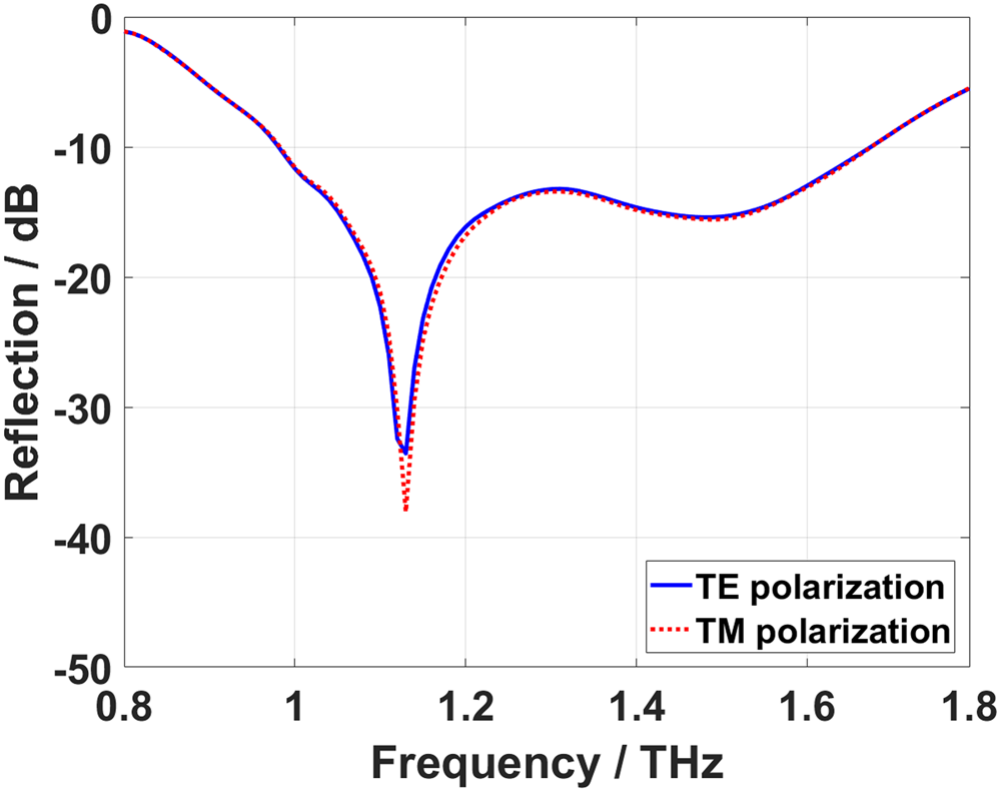
The reflection spectra of the optimal diffusion coding metasurface under TE and TM polarizations of a normal incidence.

Generally, various coding patterns of “0” and “1” elements cause digital metasurfaces to be widely utilized in masterly manipulating of the EM scattering patterns. As the main capability of the proposed graphene-based coding metasurface, real-time controlling the electrostatic bias across each of coding particles results in instant switch among different possible coding patterns, providing diverse scattering patterns with single, two, four and numerous beams only by one versatile structure. Through proper spatial mixtures of graphene-based metasurface bits, metasurfaces bytes are constructed, resulting in diverse functionalities. Here, five distinct coding patterns containing different average information quantities are evaluated as the most popular representatives of other metasurface layouts. It is expected that they react differently to the incoming EM waves. An x-polarized plane wave normally impinges on differently encoded metasurfaces and the corresponding 3D far-field patterns and in- plane patterns at f = 1.32 THz are shown in Fig. 7(I–V). The simplest coding sequence of 11…1/11…1 or 00…0/00…0 in which all metasurface bits have identical reflection phases inevitably reflects the incoming EM waves toward the normal direction, as can be seen in Fig. 7(I), imitating the perfect electric or magnetic conductor. It is obvious that a little information is contained in such a far-field pattern, as previously given in Table 1. Configuring the graphene-based metasurface with the periodic coding sequence of 1010…/1010… anomalously deflects the illuminating wave to two symmetrically oriented beams pointing to angles θ = ±27.4°, φ = 0° with respect to z- and x-axes, respectively (see Fig. 7(II) and Supplementary Information G). As another case and under the periodic coding sequence of 1010…/0101…, the normally incident wave is split into four symmetrically oriented reflected beams with the angles of θ = 41.3°, φ = 45°, 135°, 225°, 315°, as monitored in Fig. 7(III). The results clearly indicate that more information is contained in the far-field pattern of such coding patterns, and are well consistent with those previously discussed and displayed in Fig. 3c. Based on the achieved optimization-based and convolutionally encrypted diffusion layouts, the related coding metasurfaces are built, normally illuminated, and their 3D far-field patterns and in-plane patterns are given in Fig. 7(IV), (V). As can be observed, both metasurfaces are capable of quasi-isotropic scattering caused by incoming wave deflection into numerous parity beams. With a significant reduction of design complexity, the convolutionally encoded diffusion metasurface almost remains the high performances of the optimized one. Accordingly, the proposed strategy enables a simplified design method where the encoded layout is strongly random enough to play the role of a diffusion coding metasurface. To investigate the low scattering property, the reflection spectra of the optimal diffusion metasurfaces are simulated under illumination of both TE- and TM-polarized normal incidences. As illustrated in Fig. 8, low reflectivity below −10 dB is preserved in a broad frequency range from 1 THz to 1.7 THz (about 52% relative to the center frequency), in which the maximum specular reflection reduction is as high as 35 dB. Meanwhile, the TE and TM reflection spectra fully overlap each other, indicating the polarization- insensitive feature of the coding metasurfaces to be originated from the geometrical symmetry of graphene-based meta-atoms. The reflection spectra of the convolutionally encoded metasurface are also plotted in Supplementary Information H. As the key concept of this paper, the computational burden associated with the conventional optimization-based design algorithms is dramatically reduced at the expense of a negligible amount of degradation in the scattering diffusion performance. A more accurate comparison between the convolutionally encrypted and optimal diffusion metasurfaces of arbitrarily large size based on a rigorous criterion, bistatic figure of merit, is given in Supplementary Information I.

## Discussion

The new concept of encrypted coding metasurfaces was proposed in which the information of messages can be illusory recorded on multiple parity beams. To make the radiated pattern difficult to be damaged, altered or reverse-engineered, a new rapid, straightforward and efficient design fashion yielding diffusion metasurfaces was also investigated. Such phase-encrypted metasurfaces are believed to have great potential use in various security applications, optical security, verification systems and encrypted holograms. Compared with optimal diffusion layouts governed by the conventional optimization-based algorithms, the proposed design strategy dramatically reduces the time consumption and the required memory storage, while preserving the diffusion performance, convincingly. The encrypted coding patterns were realized by reconfigurable graphene-based digital building blocks whose operational status can be dynamically switched between two states of “0” and “1”. The presented study may revolutionary introduce a new route to achieve transparent, conformal and re-programmable coding metasurfaces to be utilized in real-time beam manipulation of the THz waves.

## Method

An infinitesimally-thin and anisotropic sheet specified with a certain complex surface conductivity can theoretically represent the graphene monolayer. In this case, using the well-known Kubo formula, the complex surface conductivity of graphene can be derived as the sum of the interband and intraband transition contributions^43,44^

6-a $${\sigma }_{d}(\omega )={\sigma }_{d}^{intra}(\omega )+{\sigma }_{d}^{inter}(\omega )$$

6-b $${\sigma }_{d}^{intra}(\omega )=-j\frac{{e}^{2}{k}_{B}T}{{\rm{\Pi }}{\hslash }^{2}(\omega -j2{\rm{\Gamma }})}[\frac{{\mu }_{c}}{{k}_{B}T}+2\,\mathrm{ln}({e}^{-\frac{{\mu }_{c}}{{k}_{B}T}}+1)]$$

6-c $${\sigma }_{d}^{inter}(\omega )\approx -j\frac{{e}^{2}}{4{\rm{\Pi }}\hslash }\,\mathrm{ln}\,\begin{array}{c}[\frac{2|{\mu }_{c}|-(\omega -j2{\rm{\Gamma }})\hslash }{2|{\mu }_{c}|+(\omega -j2{\rm{\Gamma }})\hslash }]\end{array}$$

in which *ω* is the operating frequency, μ_C_ is the chemical potential, Γ = 1/2τ is the phenomenological scattering rate, *τ* is the electron-phonon relaxation time, *T* is the environmental temperature, *e* = 1.6 × 10^−19^ *C* is the electron charge, k_B_ is the Boltzmann’s constant, and $\hslash =h/2\pi$ is the reduced Planck’s constant. T = 300 K and τ = 1 ps are assumed and kept constant through this paper. Here, the intraband conductivity has a familiar Drude-like dispersion form. In addition, regarding the Pauli exclusion principle^45^, the interband contribution of graphene conductivity in which we assume ${k}_{B}T\ll |{\mu }_{c}|,\,\hslash \omega$ can be neglected on account of the photon energy $\hslash \omega \ll {E}_{f}$, ${E}_{f}\gg {k}_{B}T$ in the low THz frequency region. Thanks to the tunable performance of graphene, additional flexibility in the design of various functionalities can be obtained through endowing the surface with a proper spatial distribution of chemical potentials.

## Electronic supplementary material

Supplementary Information
