# Publisher Correction: An Information Theory-Inspired Strategy for Design of Re-programmable Encrypted Graphene-based Coding Metasurfaces at Terahertz Frequencies

**DOI:** 10.1038/s41598-021-82769-1

**Published:** 2021-02-03

**Authors:** Ali Momeni, Kasra Rouhi, Hamid Rajabalipanah, Ali Abdolali

**Affiliations:** 1 Department of Electrical Engineering, Iran University of Science and Technology, Tehran, 1684613114 Iran; 2 Applied Electromagnetic Laboratory, School of Electrical Engineering, Iran University of Science and Technology, Tehran, 1684613114 Iran

Correction to: *Scientific Reports* https://doi.org/10.1038/s41598-018-24553-2, published online 18 April 2018

The Supplementary Information file that accompanies this Article contains technical errors, where mathematical symbols and equations are not displayed correctly.

The correct Supplementary Information file is linked to this correction notice.

## Supplementary information

Supplementary information.

