# Supplementary material for: Publisher Correction: An Information Theory-Inspired Strategy for Design of Re-programmable Encrypted Graphene-based Coding Metasurfaces at Terahertz Frequencies
Source: Sci Rep. 2021 Feb 3;11:3400. doi: 10.1038/s41598-021-82769-1 (PMC7859391; doi:10.1038/s41598-021-82769-1)
Supplement: Supplementary file 1 — Supplementary information. [file 41598_2021_82769_MOESM1_ESM.docx]

Supplementary Information for

**An Information Theory-Inspired Strategy for Design of Re-programmable Encrypted Graphene-based Coding Metasurfaces at Terahertz Frequencies**

**Ali Momeni**1,2**, Kasra Rouhi**1,2**, Hamid Rajabalipanah**1,2**, and Ali Abdolali**1,2, *

1 Department of Electrical Engineering, Iran University of Science and Technology, Tehran, 1684613114, Iran

2 Applied Electromagnetic Laboratory, School of Electrical Engineering, Iran University of Science and Technology, Tehran, 1684613114, Iran

[*abdolali@iust.ac.ir](mailto:*abdolali@iust.ac.ir)

**The Supplementary file includes:**

1. 2D Inverse fast Fourier transform
2. Statistical Study of the far-field patterns
3. Reflection-type information systems implemented by coding metasurfaces
4. The generator polynomials
5. Binary Bat Optimization Algorithm
6. Electrostatic bias of digital lattices
7. Cartesian far-field patterns
8. Reflection Spectra
9. Merit function for diffusion performance

**Supplementary Appendix A (2D Inverse fast Fourier transform)**

The total scattered fields of an arbitrarily arranged coding metasurface can be expressed by:

| $E_{scatt}\left( \theta,\varphi\right)=\sum_{p=1}^{N} \sum_{q=1}^{N} a_{pq}\exp\left[ jk_{0}D\left( p-1 \right)sin\theta cos\varphi\right]\exp\left[ jk_{0}D\left( q-1 \right)sin\theta sin\varphi\right]$ | (S-1) |
| --- | --- |

In u=sinθcosφ, v=sinθsinφ coordinates, the above equation turns to:

| $E_{scatt}\left( u,v \right)=\sum_{p=1}^{N} \sum_{q=1}^{N} a_{pq}\exp\left( \frac{j2\pi pD}{\lambda_{0}}u \right) \exp\left( \frac{j2\pi qD}{\lambda_{0}}v \right)$ | (S-2) |
| --- | --- |

On the other hand, with performing a 2D inverse Fourier transform of K_fft_×L_fft_ points on the complex reflection coefficients of the graphene-based lattices:

| $IFFT\left( k,l \right)=\frac{1}{K_{fft}\times L_{fft}}\sum_{p=1}^{N} \sum_{q=1}^{N} a_{pq}\exp\left( j2\pi p\frac{k}{K_{fft}} \right) \exp\left( j2\pi q\frac{l}{L_{fft}} \right);$  $k=0,1,\ldots,K_{fft}, l=0,1,\ldots, L_{fft}$ | (S-3) |
| --- | --- |

Regarding the existing duality between Eqs. (2), (3), one can represent the scattered fields of metasurface based on 2D-IFFT as:

| $E_{scatt}\left( u,v \right)=K_{fft}\times L_{fft}\times IFFT(k,l)$ | (S-4) |
| --- | --- |

Here, $\frac{D}{\lambda_{0}}u=\frac{k}{K_{fft}}+S_{k}$ and $\frac{D}{\lambda_{0}}v=\frac{l}{L_{fft}}+S_{l}$ in which, S_k_ and S_l_ are integers utilized to guarantee that both sides have the same value range. In addition, the visible region of (u,v) coordinates is determined by $u^{2}+v^{2}\leq1.$

**Supplementary Appendix B (Statistical Study of the far-field patterns)**

The 2D far-field images resulted by the 2D-IFFT technique have a stochastic behavior and form a pseudorandom scattering matrix (S), comprising the far-field pattern intensities. All the gray levels of the far-field pixels are normalized to the range of 0 and 128 and then discretized to a 7-bit integer, forming the quantized scattering matrix ($\tilde{S}_{ij}$). In this section, we are seeking to find those of measurable random variables, which adequately address the stochastic specifications of $\tilde{S}_{ij}$ components through evaluating their PDF_JP_ diagrams. Keeping this issue in mind, we define J_R_(i,j) and J_D_(i,j) as the horizontal and vertical random variables to generally indicate 2^7^×2^7^ different possible outcomes of far-field intensities for a pair of neighboring $\tilde{S}_{ij}$ elements of the right and down sides, respectively (see Figure 2). The probability of appearance for each state would reveal useful information about the statistical features of a metasurface and its far-field pattern. On the account of $\tilde{S}_{ij}$ elements, a graphical representation of the distribution of the horizontal and vertical random variables −called as histogram− helps us to determine PDF_JP_ of the far-field patterns. To construct the required histogram, the $\tilde{S}_{ij}$ matrix is entirely searched to count how many adjacent lattices on the right or down sides possess one of the quantized values, where the horizontal axis denotes the possible outcomes of the random variables. Finally, the normalized values of horizontal or vertical random variables specify the joint probability of a group J_R/D_(i,j): the probability that its value falls in any given interval (the gray level i of the current pixel and the gray level j of its adjacent pixel). The PDF_JR_ diagrams of the far-field patterns for differently arranged coding metasurfaces are depicted in Supplementary Figure S1.

**
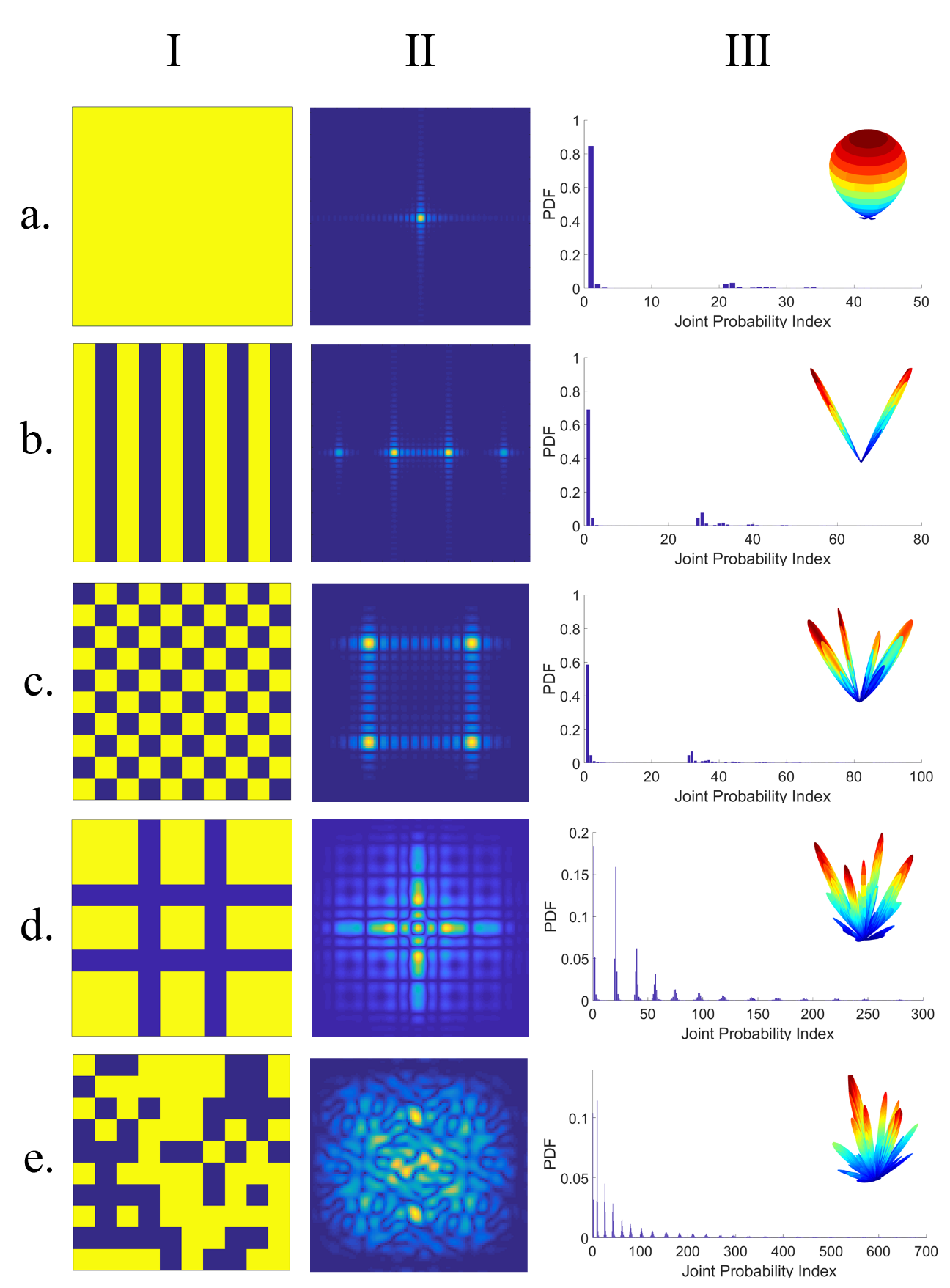
**

(I)

(II)

(III)

**Supplementary Figure S1. Differently arranged coding metasurfaces and their 2D far-field images and PDF_JP_ diagrams.** (a) 11…1/11…1 (A-type or PEC reflector). (b) 1010…/1010… (B-type or stripped configuration). (c) 1010…/0101… (C-type or chessboard configuration). **(d)** Non-periodic configuration (D-type). **(e)** Arbitrary computer-generated pseudorandom configuration (E-type). (i) Coding layouts. (ii) 2D far-field images. (iii) PDF_JR_ diagrams of the far-field images.

For a PEC mirror, the angular dependency of the far-field pattern can be represented by a single-peak 2D sinc function. Therefore, as depicted in Figure 3a, the corresponding PDF_JD_ diagram has a small number of sub-Gaussian and super-Gaussian components but with strong amplitudes. In the periodic striped and chessboard configurations, a superposition of several nonconcentric sinc functions forms far-field patterns with two and four symmetrically oriented beams, respectively. The number of sub-Gaussian and super-Gaussian components in these cases is greater than those observed in the PDF_JD_ diagram of the PEC mirror (Figures 3b, c). In the non-periodically arranged layout (cross-shaped binary message), a more disorganized far-field pattern with five radiated beams is observed, leading to a more uniform PDF_JD_ (Figure 3d). The pseudorandom coding pattern of E-type, as a special case of non-periodic configuration, creates a far-field image with numerous emitted beams, carrying much more information than the aforementioned layouts.

**Supplementary Appendix C (Reflection-Type information systems implemented by coding metasurfaces)**


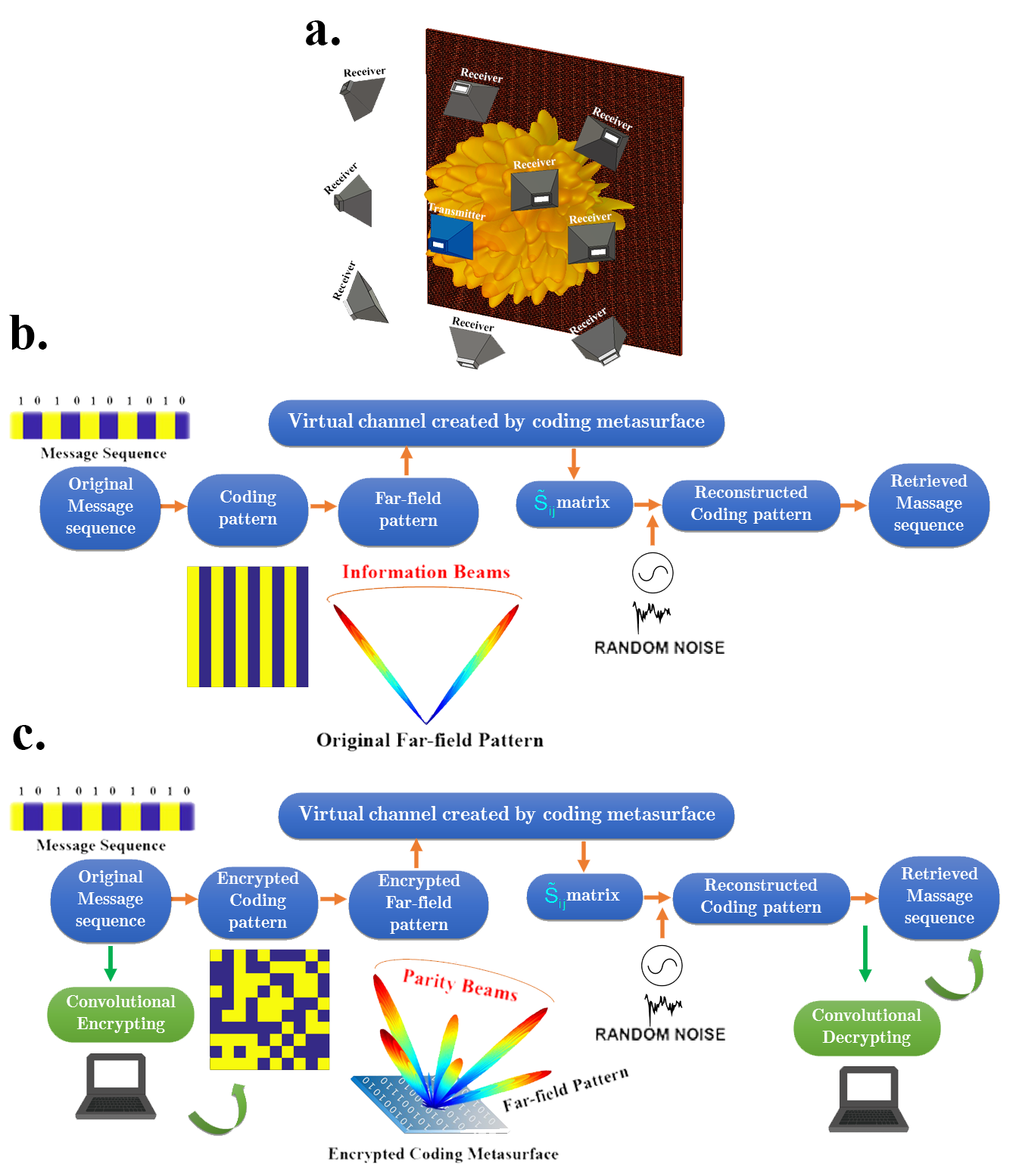


**Supplementary Figure S2.** (a) The schematic diagram of a reflection-type information system and the block diagram of digital communication in the virtual channel created by the coding metasurface itself (b) without and (c) with convolutional encryption.

**Supplementary Appendix D (The generator polynomials)**

In the general form, the established convolutional encoder of this paper has *N* inputs and *k* outputs. The octal representation of the *k* generator polynomials is first converted into the binary representation, clarifying that how the i^th^ input bit contributes to the j^th^ output bit.

| **Supplementary Table S1**. The generator polynomial utilized in the proposed convolutional phase encryption scheme (N=10, k=10) | | |
| --- | --- | --- |
| Input sequence message | Generator polynomials(g_i_) in the decimal base |  |
| 1010101010 | 25 4 7 4 7 4 7 4 7 4 |  |

| **Supplementary Table S2**. The generator polynomials utilized for designing phase-encrypted  diffusion coding patterns | | |
| --- | --- | --- |
| N | Input sequence | Generator polynomials(g_i_) in the decimal base |
| 4 | 1100 | 24 10 31 12 |
| 6 | 110101 | 31 7 27 12 14 6 |
| 10 | 1010111101 | 2 31 9 20 13 22 13 5 12 26 |
| 13 | 1101001110101 | 27 26 13 11 19 27 27 19 11 26 14 27 3 |
| 16 | 1011001110110101 | 11 7 3 8 12 18 18 24 19 5 20 4 12 10 23 4 |
| 20 | 01111010101011001010 | 13 15 12 23 20 24 28 29 7 6 21 5 17 17 26 16 13 21 23 17 |

**Supplementary Appendix E (Binary Bat Optimization Algorithm)**

One of the recently developed nature-inspired metaheuristic optimization methods for discrete global optimization is Binary Bat algorithm (BBA). According to the echolocation behavior of microbats, BBA carries the search process using artificial bats as search agents mimicking the natural pulse loudness and emission rate of real bats. As the main advantage of the BBA, it can provide a very quick convergence at a very initial stage by automatic switching from exploration to exploitation^S1^. In addition, it guarantees the global convergence properties under the right condition, and is a worthy candidate for large-scale problems as it effectively functions in such scenarios. Frequency-tuning, automatic zooming and parameter control are the key features make this optimization algorithm very efficient. As a distinct advantage over other metaheuristic algorithms, BBA is capable of automatic zooming into an area where promising solutions have been found^S2^. The autozooming ability is associated with the automatic switch from explorative moves to local intensive exploitation. The main parameters of BBA are the pulse frequency (F_i_), pulse rates (r_i_), loudness (A_i_), bat population (X_i_ and V_i_), and finally the number of iterations (N) which should be initialized before running. In contrast to the other metaheuristic algorithms, BBA can update and control the value of parameters (loudness and pulse rates) as the iterations proceed, providing a way to automatically switch between exploration and exploitation when the bats are moving toward the best solutions. BBA can be simply implemented in MATLAB where such a flexible optimization technique can be utilized to solve our optimization problem because of its distinctive features. In this paper, the 2D coding matrix of M has been randomly initialized by the 0/1 binary digits, as the bat population (X_i_). The other parameters of V_i_, r_i_, A_i_, F_i_, and N are set as 0, 0.1, 0.25, 0 and 350, respectively. To achieve the best stealth performance, we are looking to find the optimal diffusion layout of the encoded metasurface by minimizing the following merit function:

***Fitness function:*** $\min\left( 1/H_{RE} \right)=\min\left\{ -2/\log(\sum_{i=1}^{128} \sum_{i_{R}=1}^{128} P_{ii_{R}}^{2}+\sum_{i=1}^{128} \sum_{i_{D}=1}^{128} P_{ii_{D}}^{2}) \right\}$ (S-5)

in which, H_RE_ denotes the Renyi entropy value, $P_{ii_{R}}$ and $P_{ii_{D}}$refer to the elements with the indexes of ii_R_ and ii_D_ of PDF_JR_ and PDF_JD_, respectively. Supplementary Figure S3 demonstrates the flowchart of the proposed method for optimization of the far-field pattern information to achieve the optimal coding matrix, based on the BBA. For a coding metasurface of 100 elements, the total time consumption for all 350 iterations is 14400 s (4h). Compared with the initial H_RE_, the maximum Renyi entropy value has sharply increased to reach 1.7946. As a result, a quasi-uniform distribution of PDF_JP_ with a greater number of weak sub-Gaussian and super-Gaussian components has been obtained, leading to a very low scattering encoded metasurface.


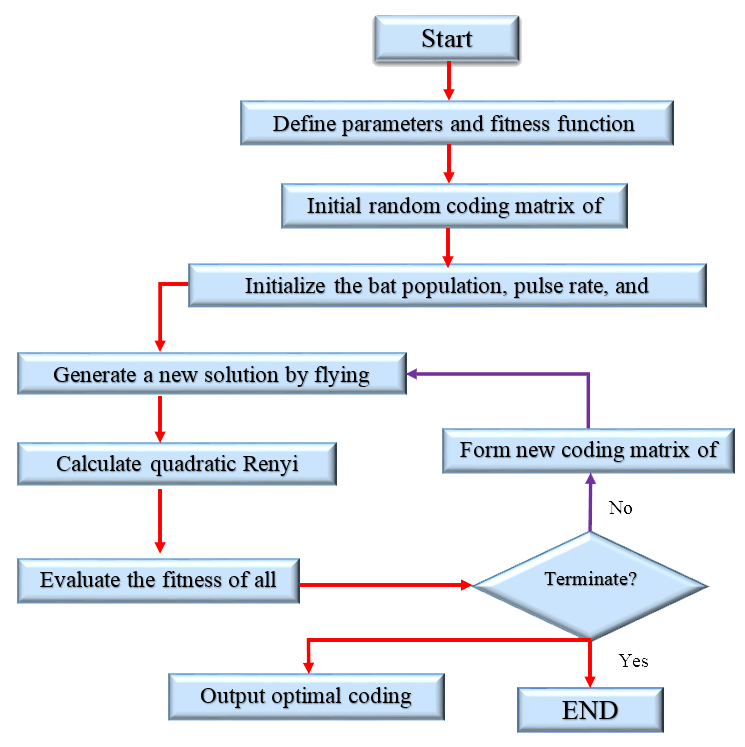


**Supplementary Figure S3.** The flowchart of the employed Binary Bat optimization algorithm for finding the optimal diffusion coding matrix.

**Supplementary Appendix F (Electrostatic bias of digital lattices)**

The exotic mechanical and optical features along with moderate losses, and the exceptional property of being tunable through external electrostatic or magnetostatic, introduce graphene as a promising platform for realizing various THz functionalities with unprecedented possibilities. The graphene surface can be externally biased with both of electrostatic and magnetostatic fields (Hall effect), allowing to dynamically control the real and imaginary parts of its surface conductivity. Based on a semi classical quantum mechanical approach, the 2D graphene surface conductivity perfectly follows the tensor form of ^S3^

| $\sigma\left( \omega,\mu_{c}\left( \boldsymbol{E}_{0} \right),\Gamma,T,\boldsymbol{B}_{\boldsymbol{0}} \right)=\boldsymbol{I}_{t}\sigma_{d}+\boldsymbol{J}_{t}\sigma_{O}$ | (S-6) |
| --- | --- |

in which ω is the operating frequency, μ_C_ is the chemical potential, Γ=1/2τ is the phenomenological scattering rate, τ is the electron-phonon relaxation time, T is the environmental temperature, and E_0_ and B_0_ are the external electrostatic and magnetostatic bias fields, respectively. Also, $\sigma_{d}$ and $\sigma_{O}$ respectively refer to diagonal and off-diagonal conductivities, and $\boldsymbol{I}_{t}=\hat{x}\hat{x}+\hat{y}\hat{y}$ and $\boldsymbol{J}_{t}=\hat{x}\hat{x}-\hat{y}\hat{y}$ are the symmetric and antisymmetric dyads, respectively. T=300 K and τ=1 ps, are assumed and kept constant through this paper. In the absence of magnetic bias, the off-diagonal elements of surface conductivity vanish and graphene behaves as an isotropic surface^S3^. In this case, using the well-known Kubo formula, the complex surface conductivity of graphene can be derived as the sum of the interband and intraband transition contributions^S3^

| $\sigma_{d}\left( \mu_{c}\left( E_{0} \right) \right)=\frac{je^{2}\left( \omega-j2\Gamma\right)}{\prod\hbar^{2}}\left[ \frac{1}{\left( \omega-j2\Gamma\right)^{2}}\int_{0}^{\infty} \varepsilon\left( \frac{\partial f_{d}\left( \varepsilon\right)}{\partial\varepsilon}-\frac{\partial f_{d}\left( -\varepsilon\right)}{\partial\varepsilon} \right)d\varepsilon-\int_{0}^{\infty} \left( \frac{f_{d}\left( -\varepsilon\right)-f_{d}(\varepsilon)}{\left( \omega-j2\Gamma\right)^{2}-4\left( \frac{\varepsilon}{\hbar} \right)^{2}} \right)d\varepsilon\right]$ | (S-7) |
| --- | --- |

where $e=1.6\times{10}^{-19}C$ is the electron charge, k_B_ is the Boltzmann’s constant, $f_{d}=\left\{ e^{\frac{\varepsilon-\mu_{c}}{k_{B}T}}+1 \right\}^{-1}$ is the Femi-Dirac distribution, and $\hbar=h/2\pi$ is the reduced Planck’s constant. The first and second terms of Eq. (S-7) are due to intraband and interband contributions, respectively, and can be evaluated as^S3^

| \| $\sigma_{d}^{intra}\left( \omega\right)=-j\frac{e^{2}k_{B}T}{\Pi\hbar^{2}\left( \omega-j2\Gamma\right)}\left[ \frac{\mu_{c}}{k_{B}T}+2\ln\left( e^{- \frac{\mu_{c}}{k_{B}T}}+1 \right) \right]$ \| (S-8-a) \| \| --- \| --- \| \| $\sigma_{d}^{inter}\left( \omega\right)\approx-j\frac{e^{2}}{4\Pi\hbar}\ln\begin{aligned} \left[ \frac{2\left\vert\mu_{c} \right\vert-\left( \omega-j2\Gamma\right)\hbar}{2\left\vert\mu_{c} \right\vert+\left( \omega-j2\Gamma\right)\hbar} \right] \\ \end{aligned}$ \| (S-8-b) \| |  |
| --- | --- | --- | --- | --- | --- |

Here, the intraband conductivity has a familiar Drude-like dispersion form. In addition, regarding the Pauli exclusion principle, the interband contribution of graphene conductivity in which we assume $k_{B}T\ll\left| \mu_{c} \right|, \hbar\omega$ can be neglected on account of the photon energy $\hbar\omega\ll E_{f}$ , $E_{f}\gg k_{B}T$ in the low THz frequency region. The surface conductivity of graphene can be set to a constant during the fabrication, chemical vapor deposition (CVD) method, or be controlled in a real-time manner using an external DC bias voltage^S4-S6^. In a bias-less graphene sheet, despite having the spatial dispersion, the graphene surface has neither electrostatic nor magnetostatic bias (E_0_=B_0_=0) and the conductivity components become operators. To achieve dynamic reconfiguration through shifting the Fermi energy level, two special solutions can be imagined to change the graphene's conductivity tensor. In the first case, referred as local Hall effect regime, an external magnetostatic bias, and possibly electrostatic bias (E_0_$\neq$0, B_0_$\neq$0) are applied to the graphene surface but with no spatial dispersion. As the second case, in the presence of external electrostatic bias (E_0_$\neq$0), no magnetostatic bias nor spatial dispersion, the graphene conductivity is a scalar. An applied electric field bias injects more electron or holes carriers in graphene and thereby allows dynamic controlling of both real and imaginary part of the conductivity at terahertz frequencies. The latter case is considered in this paper. In these methods, we always seek to control the chemical potential of graphene, which is actually realized by changing the carrier concentration. The chemical potential and the carrier concentration of graphene surface can be linked together through $\mu_{c}=\hbar v_{f}\sqrt{\pi n}$ in which n(m^-2^) denotes the carrier concentration, $\mu_{c}\left( eV \right)$ is the chemical potential, $\hbar$ is the reduced Planck's constant and finally *v_f_* refers to the Fermi velocity. Assuming the model of a capacitor (C_g_) between the graphene metasurface and the SiO_2_ semiconductor with the filler dielectric of alumina (99.5%) ($\varepsilon_{r}=9.9, tan\delta=0.0001, h)$, one can determine the existing relation between the voltage and the carrier's concentration as $n=C_{g}V_{\mathrm{Bias}}/q_{e}$. Here, *V_Bias_* is the external DC bias voltage (gate voltage) applied to the graphene-insulator-semiconductor structure and $q_{e}$ is the electron charge. Accordingly, the solution of the associated electrostatic configuration allows finding the required external voltage bias, which in turn provides graphene's chemical potential as^S5^

| $V_{Bias}=\frac{q_{e}\mu_{c}^{2}h}{\pi\hbar^{2}v_{f}^{2}\varepsilon_{0}\varepsilon_{r}}$ | (S-9) |
| --- | --- |

The Fermi energy level experiences a sufficient variation covering the region of interest while the gate voltage is reasonably changed, enabling the real-time control of the graphene conductivity. Compared with the previously reported coding metasurfaces, the operational status of each lattice in the proposed terahertz graphene-based coding metasurface can be flexibly tuned by changing its top-gate voltage. One possibility to create the desired 0/1 coding patterns across the surface is to use gate voltage and split gates locally to change the graphene's conductivity at different lattices. Indeed, applying different DC gate voltages between the graphene and the SiO_2_ substrate at separate lattices leads to a certain electrostatic field in each lattice that is different from the static field elsewhere in the coding metasurface. This allows the user to individually alter the operational status of each digital lattice in a real-time manner; thereby providing a real-time THz beam manipulation capability for the proposed coding metasurface at THz frequencies. Obviously, care should be taken that the possible switching speeds do not degrade the overall real-time manipulation performance of the coding metasurface. For this aim, a comprehensive discussion about the bias circuit and the corresponding switching speeds should be accomplished to guarantee the real-time manipulation of THz scattering patterns by the proposed GBCM. As mentioned in the paper, the proposed coding metasurface consists of 10$\times$10 digital lattices that each of them occupied by 15$\times$15 Jerusalem-type graphene sheets. Without loss of generality, a perspective view of one of the constitutive graphene-based lattices is schematically depicted in Supplementary Figure S4. As can be seen, an *Au* metallic pad is utilized to electrically touch the first column of graphene metasurfaces at each lattice, separately.


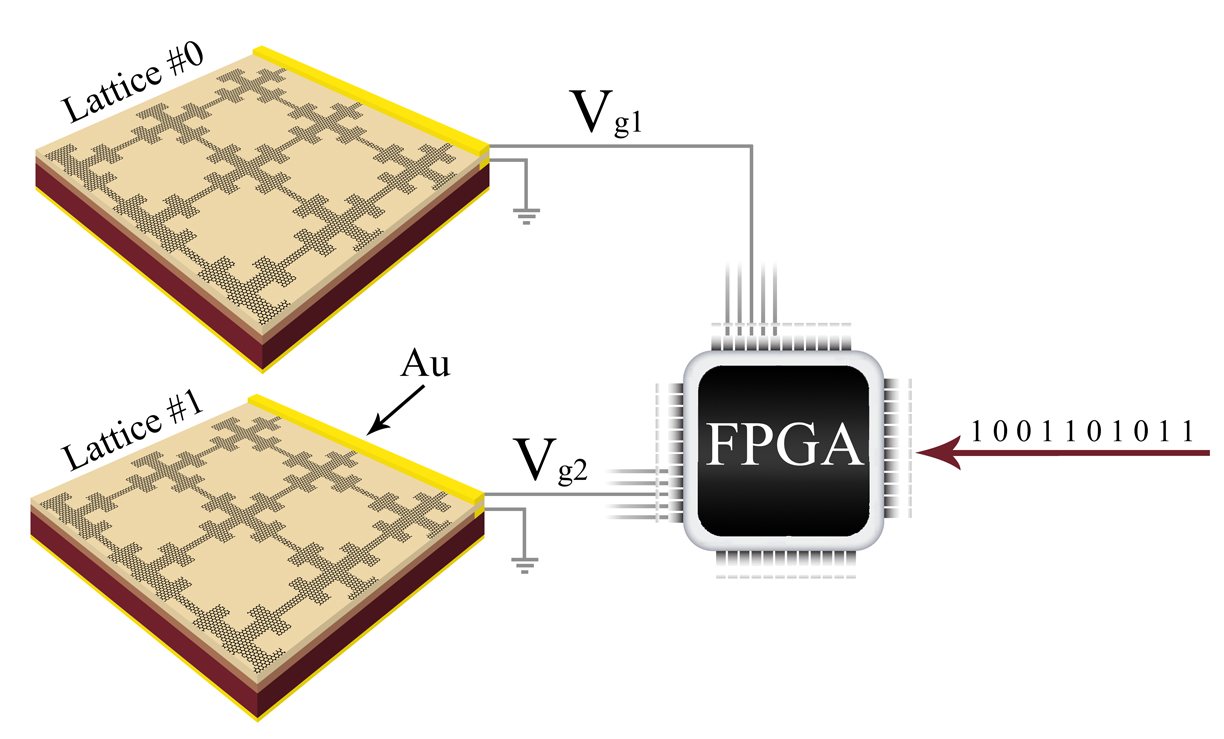


**Supplementary Figure S4.** A perspective view of dynamically switchable meta-atom of graphene-based digital particles. The structure consists of four layers: Quartz substrate, Alumina substrate, SiO_2_ substrate, and Jerusalem-shaped graphene reflective cells, wherein the gate voltage of V_g_ is utilized to dynamically switch the digital operational status of each lattice. A field programmable gate array can be utilized to independently set the operational status of each lattice.

Finally, these *Au* connections are individually linked to the output terminals of a field-programmable gate array (FPGA), as a control unit, through thin conductive bias wires^S7^. The desired 0/1 coding patterns are already registered to the internal memory of the FPGA. All the particles of a lattice are electrically linked together through deep-subwavelength graphene ribbons (to act as a top gate) whereby we can simply tune their chemical potentials with one electrostatic voltage instead of adjusting all the occupying elements, independently. Since the underlay of all lattices is a thin layer of silicon, this layer can be entirely DC grounded by contacting the zero potential *Au* pad to one side of it. Considering the Eq. S-9, the required top-gate voltages can be instantaneously delivered to each lattice using the employed FPGA, thereby determining the "ON/OFF" status of the lattices, separately. Accordingly, through a real-time switching among different pre-determined coding patterns, different scattering functionalities can be attained by utilizing the proposed coding metasurface. One of the most important factors in achieving a real-time beam manipulation is the high switching rate capability of the coding metasurface among its various functionalities, which depends on several parameters in our structure. The first factor is the speed of graphene reaction to the bias change, which is a function of relaxation time and cannot be changed after the fabrication. The associated delay time is very small and ultimately does not have a much restrictive impact on the speed of switching^S8^. On the other hand, *Au/graphene* as well as the *Au/silicon* ohmic contacts directly affect the present resistance and so the time constant ($\tau=RC$) of the bias circuit. An important way to reduce the contact resistance is to use a more efficient contact between the metallic pads and the patterned graphene sheets. Increasing the thickness of Al_2_O_3_ or establishing a low permittivity substrate instead of it can also reduce the capacitance of the bias circuit, yielding more switching speeds but at the cost of higher *V_Bias_*. It should be noted that the switching speed between different operational statuses of coding metasurface is also restricted by the operating frequency of FPGA, i.e. about a few hundred megahertz^S8^. However, this speed is still so high that one can envision a real-time THz wave manipulation for the proposed coding metasurface. Thus, the proposed idea is experimentally feasible and within the realm of current fabrication technology. All the graphene-based coding particles of "0" and "1" lattices are geometrically identical, but possess different chemical potentials. To characterize each of coding particles, the Jerusalem-shaped metasurfaces are built in CST Microwave Studio and the frequency-domain solver is adopted. Floquet ports with TE (y-polarized) and TM(x-polarized) modes are set as the excitation sources in the z-direction while the periodic boundaries are applied on the x- and y-directions. The graphene metasurface is numerically modeled by an ultrathin layer with the thickness of $\Delta=1nm$ whose relative permittivity is $\varepsilon_{r_{G}}=1+\sigma_{S}/j\omega\varepsilon_{0}\Delta$. Here, $\sigma_{S}$is the complex surface conductivity of graphene. The phase and amplitude of reflection spectra for different chemical potentials have been calculated, while the surface of the structure is chosen as the phase reference plane. The corresponding results are depicted in Supplementary Figure S5. By observing the results we find that the digital particle with two chemical potentials of $\mu_{c}=0.1 eV$ and $\mu_{c}=1.16 eV$ can act as the "0" and "1" digital particles of graphene-based coding metasurfaces. After witch, differently arranged encoded metasurfaces are constructed. Open boundaries are assigned to all directions where a plane wave normally impinges on the total structure. The time-domain solver in CST Microwave Studio is utilized to extract the resultant far-field patterns. Various functionalities governed by the graphene-based scheme show that the top-gate voltage through electric field effect can dynamically switch the operational status of each lattice; thereby allows us to manipulate the terahertz waves, instantaneously.


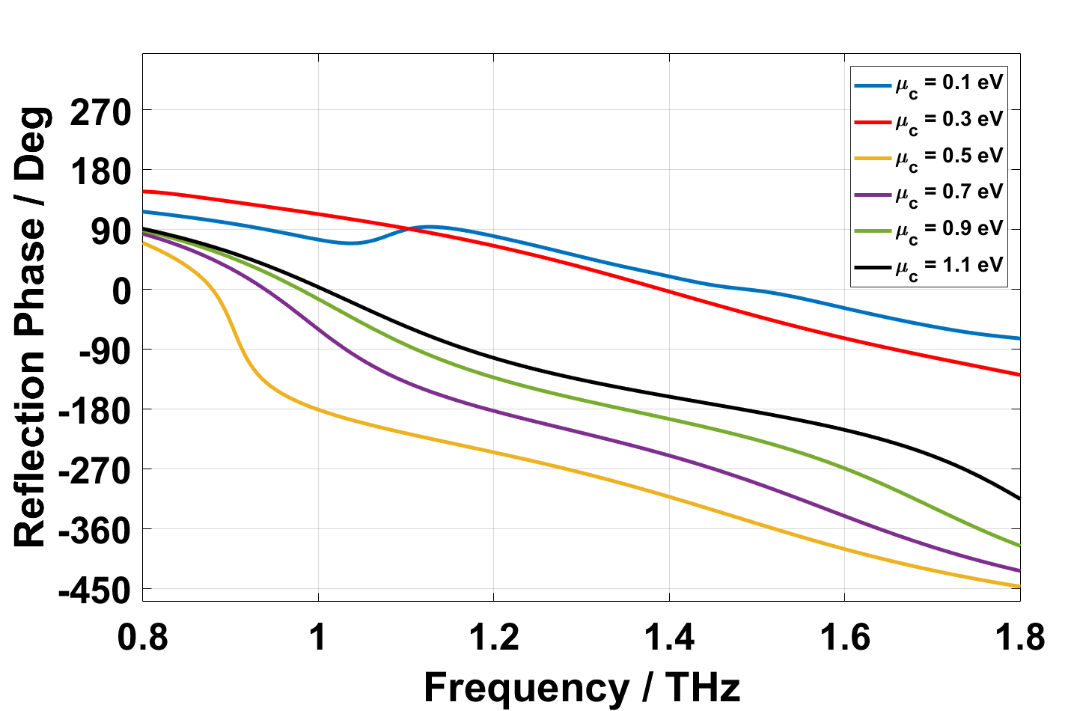


**Supplementary Figure S5.** The reflection phase spectra of the graphene-based reflective cells under different chemical potentials.

**Supplementary Appendix G (Cartesian far-field patterns)**

In addition, the simulated angular directions of scattered beams are in a good agreement with theoretically predicted ones based on the generalized Snell’s law. A reflection phase gradient of $\pi/p$ along the horizontal or vertical direction would redirect the normal incidence to the angles of θ_ref_ = ± arcsin(λ_0_/Γ) ≅ ±32.6°, in which λ_0_ is the operating wavelength and Γ is the periodicity of the phase gradient. Endowing the metasurface with a phase gradient of π/p along both the horizontal and vertical directions reflects the incoming wave to the directions of θ_ref_ = ± arcsin(√2 λ_0_/Γ) ≅ ±49.6°, (see Supplementary Figure S6).

**
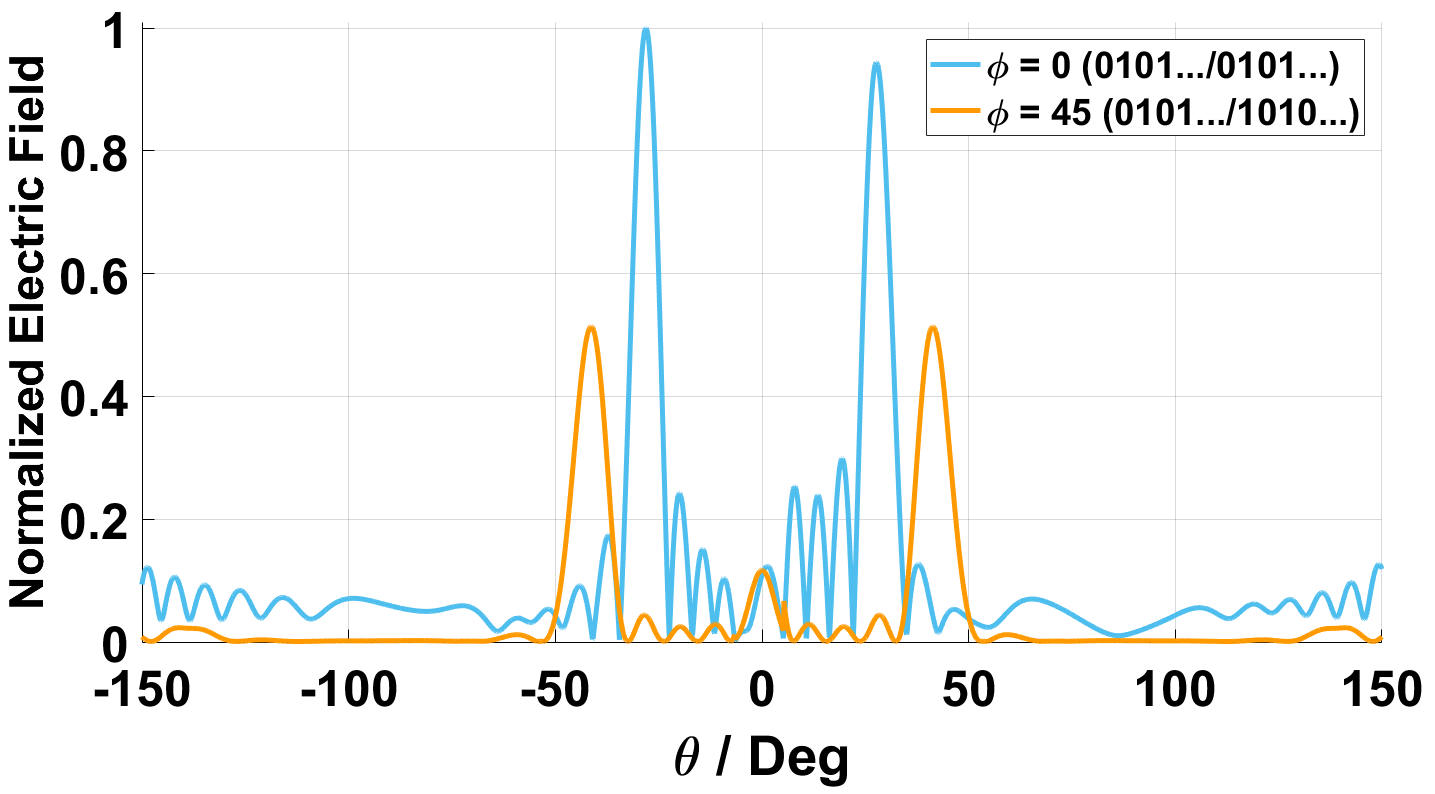
**

**Supplementary Figure S6.** The Cartesian far-field patterns of coding metasurfaces with different coding sequences of 0101…/0101… and 0101…/1010….

**Supplementary Appendix H (Reflection Spectra)**

**
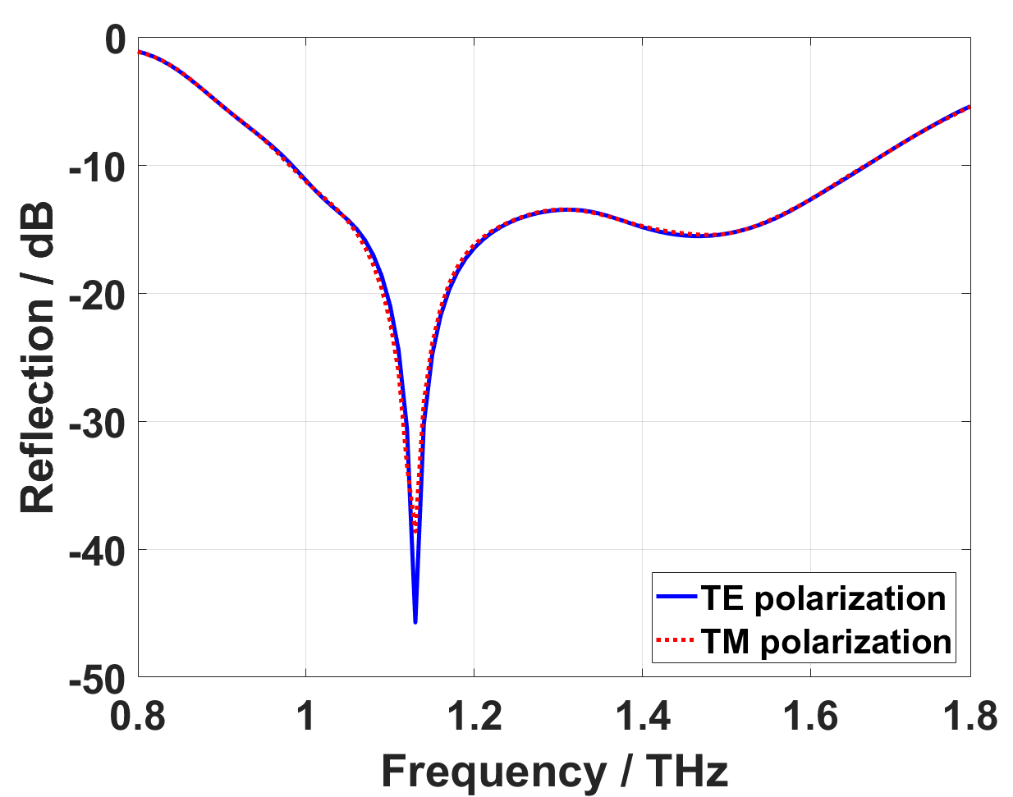
**

**Supplementary Figure S7.** The reflection spectra of the convolutionally encoded metasurface under TE and TM polarizations of a normal incidence.

**Supplementary Appendix I (Merit function for diffusion performance)**

To compare the diffusion performance of the convolutionally and optimally encoded diffusion metasurfaces, quantitatively, a figure of merit is defined as:

| $\mathrm{FMB}\left( f \right)=\frac{\mathrm{Max}\left[ Bistatic reflection of the encoded metasurface \right]}{\mathrm{Max}\left[ Bistatic reflection of the reference metallic plate \right]}$ | (S-10) |
| --- | --- |

in which, the Max{.} function domain covers all bistatic scattering angles $(0\leq\theta_{S}\leq\pi/2, 0\leq\varphi_{S}\leq2\pi)$ for a given frequency and incident angle of$(\theta_{i},\varphi_{i})$. Based on the energy conversion principle, by deflecting the incoming electromagnetic waves to numerous directions, the amount of FMB has a significant reduction compared with the reference metallic plate of the same size. Actually, as the reflected power is distributed more uniformly toward the upper half-space, the bistatic scattering pattern has a smaller maximum value.


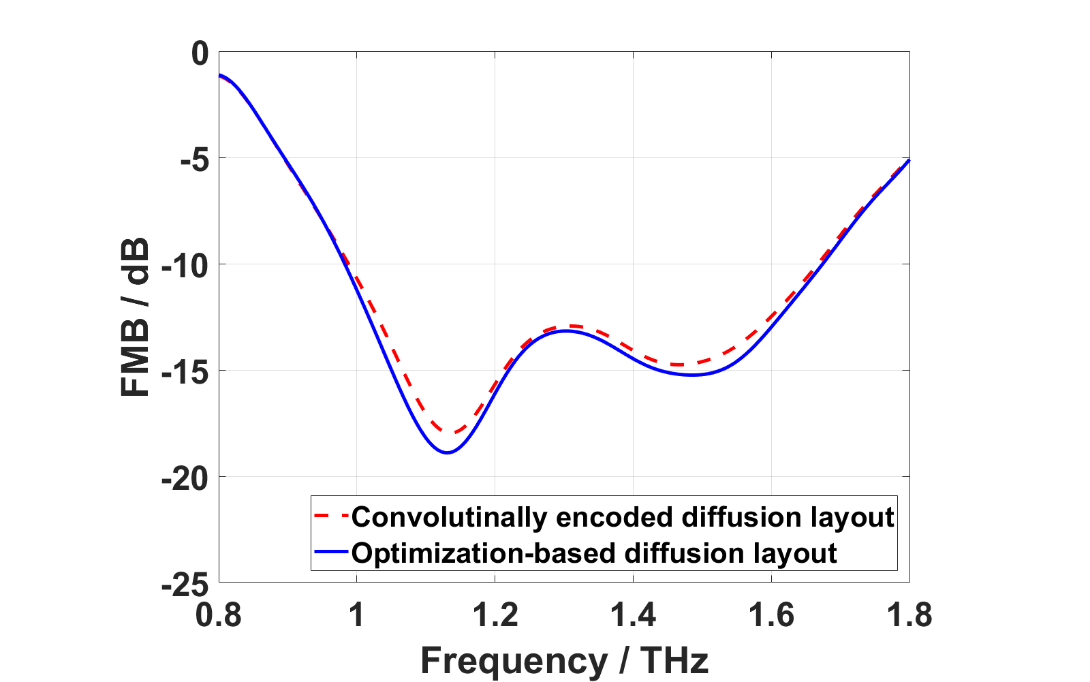


**Supplementary Figure S8.** A quantitative comparison between the scattering diffusion performance of the convolutionally encoded and the optimization-based diffusion layouts

The FMB values extracted from the corresponding 3D far-field patterns of Figures 6 (IV), (V) are plotted in Supplementary Figure S8. A metallic plate with the same size of the coding metasurface is utilized as the reference. As can be seen, the power of the reflected beams is strongly suppressed by more than 10 dB compared with the reference metallic plate in the frequency band of interest. In addition, the randomness of convolutionally encoded metasurface is somehow that it convincingly mimics the bistatic scattering behavior of the optimal diffusion metasurface. This issue highlights the capability of the proposed rapid design strategy to achieve highly efficient diffusion metasurfaces. Therefore, the low scattering property of the metasurface is essentially attributed to the scattering diffusion phenomenon. Here, based on the defined FMB measure, a fair comparison between the obtained FMB values and the lowest possible values of FMB governed by a semi-analytical prediction (as a benchmark, FMB_min_) will be made to more illustrate the capability of the proposed convolutional encryption strategy for different metasurface sizes. To achieve this, Equation (S-10) can be re-expressed as below^S9^

| $FMB\left( f \right)\approx\frac{c^{2}}{N^{2}D^{2}f^{2}}max\left( \frac{{\left\vert E_{\mathrm{elem}}\left( \theta,\varphi\right) \right\vert^{2} \left\vert\mathrm{AF}\left( \theta,\varphi\right) \right\vert}^{2}}{\int_{0}^{\frac{\pi}{2}} \int_{0}^{2\pi} {\left\vert E_{\mathrm{elem}}\left( \theta,\varphi\right) \right\vert^{2} \left\vert\mathrm{AF}\left( \theta,\varphi\right) \right\vert}^{2}sin\theta d\theta d\varphi} \right)_{\theta,\varphi}$ | (S-11) |
| --- | --- |

Based on the above formula, a semi-perfect isotropic scattering can be imagined when the array factor becomes constant throughout the upper half-space, i.e. $\left| \mathrm{AF}\left( \theta,\varphi\right) \right|=c\mathrm{te}$. In this case, the lower bound of FMB level can be approximately written as:

| $FMB_{min}(\theta,\varphi)\approx\frac{c^{2}}{N^{2}D^{2}f^{2}}max\left( \frac{\left\vert E_{\mathrm{elem}}\left( \theta,\varphi\right) \right\vert^{2}}{\int_{0}^{\frac{\pi}{2}} \int_{0}^{2\pi} \left\vert E_{\mathrm{elem}}\left( \theta,\varphi\right) \right\vert^{2}sin\theta d\theta d\varphi} \right)_{\theta,\varphi}$ | (S-12) |
| --- | --- |

The FMB_min_ is the optimal value of bistatic scattering reduction could be obtained via an optimized diffusion surface of N^2^ supercells, leading to an isotropic far-field pattern. Moreover, the far-field pattern function of elements can be assumed as:

| $\left\vert E_{\mathrm{elem}}\left( \theta,\varphi\right) \right\vert^{2}=sinc^{2}\left( 0.5k_{x}D \right)\sin c^{2}\left( 0.5k_{y}D \right)\left( \sin^{2} \theta sin\varphi+\cos^{2} \theta\right)$ | (S-13) |
| --- | --- |

where, $k_{x}=ksin\theta cos\varphi$ and $k_{y}=ksin\theta sin\varphi$ indicate the x-, and y-directed wavenumbers, respectively. Returning to Equation (S-12), one can find the FMB_min_ level of a metasurface of N^2^ elements by:

| $FMB_{min,dB}(N)\approx-20\log N-10\log\varsigma$ | (S-14) |
| --- | --- |

**
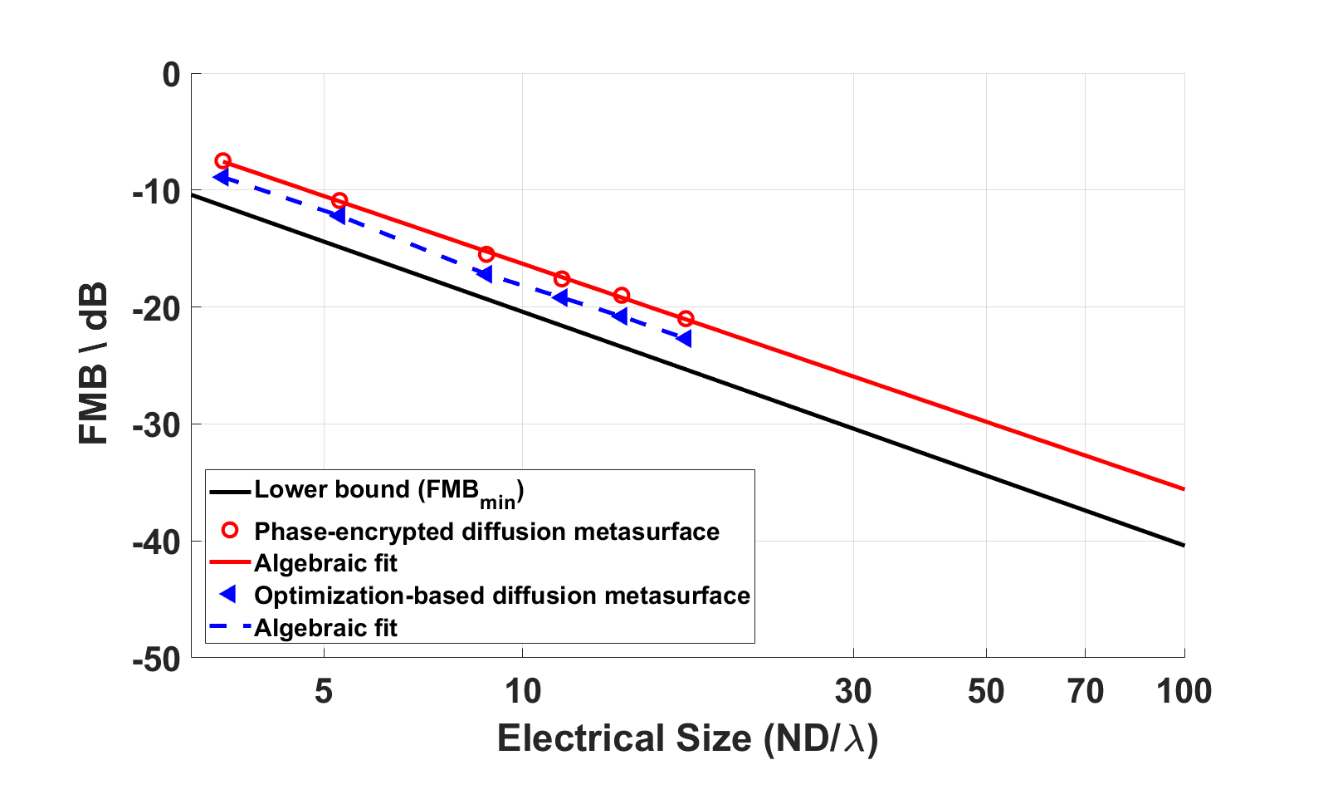

Supplementary Figure S9.** A comparison between the FMB_min_ level and the FMB values of: the numerically optimized, and the convolutionally encrypted diffusion coding patterns.

Herein, we assume $\xi=max\left( \left| E_{\mathrm{elem}}\left( \theta,\varphi\right) \right|^{2}/\int_{0}^{\frac{\pi}{2}} \int_{0}^{2\pi} \left| E_{\mathrm{elem}}\left( \theta,\varphi\right) \right|^{2}sin\theta d\theta d\varphi\right)_{\theta,\varphi}$ and
 $D\approx\lambda$. As can be seen in Figure S8 (log-log scale), a linear decreasing trend is attained in which $-10\log\varsigma$ is an offset value irrespective of the coding metasurface size. The FMB_min_ (N) levels are computed for diffusion metasurfaces of arbitrarily size and are shown in Supplementary Figure S9. To give more intuition about the results, the optimal FMB values obtained by the optimized diffusion layouts are depicted in the same figure. Obviously, due to extreme computational complexities associated with such optimization-based design algorithms and considering our computational resources, we only have the ability to provide the optimization results for N<20. A negligible difference (about 2dB) between the FMB values of the optimized diffusion layouts and the FMB_min_ (N) allows us to reliably establish the semi-analytical predictions of Equation (S-14), as a benchmark, to evaluate the performance of our designed diffusion metasurfaces of arbitrarily large size. A small discrepancy between the results can also be attributed to the limited number of spectral-shaping capabilities expected from different distributions of 1-bit binary reflection phases.

As previously mentioned, while preserving a convincing diffusion performance, based on the convolutional encryption scheme, this paper offers a fast strategy for real-time programming of very large diffusion metasurfaces. To better convey design intent, the FMB values achieved by the convolutionally encrypted diffusion metasurfaces (4<N<100) are also given in the same Figure S8. An acceptable agreement (very similar slopes and only a few dB differences) between the semi-analytical lower bound of FMB (FMB_min_), the FMB values assigned to the numerically optimized layouts in the full-wave simulations, and the FMB quantities achieved by our phase-encrypted diffusion metasurfaces is observable. As a result, the phase-encrypted diffusion metasurfaces of this paper exhibit a convincing and comparable diffusion level while they are designed in a faster and simpler manner. Indeed, as an interesting connection with the digital communication, the capability of the convolutional encryption scheme is borrowed as a reliable design tool for arbitrarily large diffusion metasurfaces where the other design approaches are computationally unaffordable.

**References**

S1. Mirjalili, S., Mirjalili, S. M. & Yang, X. S. Binary bat algorithm. *Neural Comput. Appl.* **25,** 663–681 (2014).

S2. Yang, X.-S. Bat Algorithm: Literature Review and Applications. *Int. J. Bio-Inspired Comput.* **5,** 141–149 (2013).

S3. Hanson, G. W. Dyadic green’s functions for an anisotropic, non-local model of biased graphene. *IEEE Trans. Antennas Propag.* **56,** 747–757 (2008).

S4. Sherrott, M. C. *et al.* Experimental Demonstration of >230° Phase Modulation in Gate-Tunable Graphene–Gold Reconfigurable Mid-Infrared Metasurfaces. *Nano Lett.* **17,** 3027–3034 (2017).

S5. Gao, W. *et al.* High-contrast terahertz wave modulation by gated graphene enhanced by extraordinary transmission through ring apertures. *Nano Lett.* **14,** 1242–1248 (2014).

S6. Liu, M. *et al.* A graphene-based broadband optical modulator. *Nature* **474,** 64–67 (2011).

S7. Yunhong Ding *et al.* Effective Electro-Optical Modulation with High Extinction Ratio by a Graphene–Silicon Microring Resonator. *Nano Lett.* **15,** 4393–4400 (2015).

S8. Dabidian, N. *et al.* Experimental demonstration of phase modulation and motion sensing using graphene-integrated metasurfaces. *Nano Lett.* **16,** 3607–3615 (2016).

S9. Cui, T. J., Qi, M., Wan, X., Zhao, J. & Cheng, Q. Coding Metamaterials, Digital Metamaterials and Programming Metamaterials. *Light Sci. Appl.* **3,** e218 (2014).
